# Deep learning detection of dynamic exocytosis events in fluorescence TIRF microscopy

**DOI:** 10.1371/journal.pcbi.1013556

**Published:** 2025-10-14

**Authors:** Hugo Lachuer, Emmanuel Moebel, Anne-Sophie Macé, Arthur Masson, Kristine Schauer, Charles Kervrann

**Affiliations:** 1 Université Paris Cité, CNRS, Institut Jacques Monod, Paris, France; 2 Inria Center at University of Rennes, SAIRPICO Team, U1339 INSERM, Institut Curie, Chemical Biology of Cancer Unit, Paris, France; 3 Institut Curie, PSL Research University, CNRS, UMR 144, Cell and Tissue Imaging Facility (PICT-IBiSA), Paris, France; 4 Université Paris-Saclay, Gustave Roussy, Inserm U1279, Centre National de la Recherche Scientifique, Villejuif, France; University of Zurich Faculty of Mathematics and Science: Universitat Zurich Mathematisch-Naturwissenschaftliche Fakultat, SWITZERLAND

## Abstract

Segmentation and detection of biological objects in fluorescence microscopy is of paramount importance in cell imaging. Deep learning approaches have recently shown promise to advance, automatize and accelerate analysis. However, most of the interest has been given to the segmentation of static objects of 2D/3D images whereas the segmentation of dynamic processes obtained from time-lapse acquisitions has been less explored. Here we adapted DeepFinder, a U-Net originally designed for 3D noisy cryo-electron tomography (cryo-ET) data, for the detection of rare dynamic exocytosis events (termed ExoDeepFinder) observed in temporal series of 2D Total Internal Reflection Fluorescence Microscopy (TIRFM) images. ExoDeepFinder achieved good absolute performances with a relatively small training dataset of 12000 events in 60 cells. We rigorously compared deep learning performances with unsupervised conventional methods from the literature. ExoDeepFinder outcompeted the tested methods, but also exhibited a greater plasticity to the experimental conditions when tested under drug treatments and after changes in cell line or imaged reporter. This robustness to unseen experimental conditions did not require re-training demonstrating generalization capability of our deep learning model. ExoDeepFinder, as well as the annotated training datasets, were made transparent and available through an open-source software as well as a Napari plugin and can directly be applied to custom user data. The apparent plasticity and performances of ExoDeepFinder to detect dynamic events open new opportunities for future deep learning guided analysis of dynamic processes in live-cell imaging.

## Author summary

Until now, deep learning in microscopy has been largely restricted to the detection of static biological objects. Here, we adapted a neural network designed for macromolecule detection in 3D electron microscopy to instead detect dynamic exocytosis events from time-lapse fluorescence microscopy, calling it “ExoDeepFinder”. Exocytosis detection is of prime interest because of its involvement in many fundamental physiological processes, from digestive enzyme secretion to neurotransmitter release. Comparing ExoDeepFinder to traditional exocytosis detection methods on a large dataset, we demonstrated the superiority of our deep learning approach. ExoDeepFinder is more robust than other methods, even when faced with experimental conditions unseen during training. Our dataset and method are transparent and freely available, thus enabling re-training and extension for other dynamic cell processes.

## Introduction

**Fig 1:**
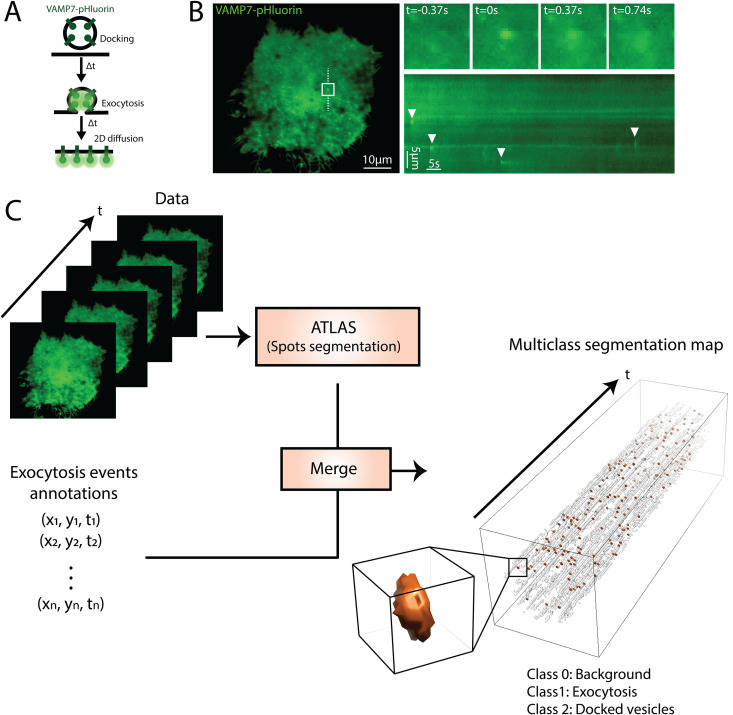
A. Schematic representation of the exocytosis of a VAMP7-pHluorin-positive vesicle: the low pH of the acidic lumen quenches the fluorescence of pHluorin. During exocytosis, protons are released and pHluorin starts to emit light. An exocytosis event is followed by the 2D diffusion of VAMP7-pHluorin at the plasma membrane. B. TIRFM image of VAMP7-pHluorin in a transfected RPE-1 cell. The inset represents the field in the white square showing one exocytosis event at different time points, *t*  = 0 represents the peak of the exocytosis event. A kymograph is plotted along the dashed white line and arrowheads indicate several observed exocytosis events. C. Workflow of the data preparation for ExoDeepFinder training. On the one hand, manual annotation gives the coordinates of ‘ground truth’ exocytosis events, on the other hand, spots corresponding to docked vesicle are detected thanks to the ATLAS algorithm. Both annotations are merged to produce a multiclass segmentation map. The resulting spatial coordinates are converted into a 3D mask. The luminescence of an exocytosis event is isotropic in the (*x,y*) plane and has an exponential decay in time. Therefore, we model an exocytosis event as a tube with an exponentially decaying radius starting at R = 4 pixels and ending at R = 1 pixel, the length of the tube being 3 frames in the temporal dimension as illustrated in the insight in orange. Hence, our segmentation map is composed of 3 classes: background (class 0, not depicted), *bona fide* exocytosis event (class 1, orange) and docked vesicles/constant spot detected by ATLAS (class 2, gray).

Technological improvements in imaging accelerate the amount of acquired data. This situation requires new methods to automatically extract the tremendous quantity of information present in them. It is clearly established that deep learning-based image segmentation methods, and especially U-Net [1], surpass conventional techniques and exhibit a remarkable generalisation capacity [1,2]. However, most of the studies are restricted to the segmentation of static biological objects. Deep learning-methods are less regularly applied to dynamic processes in biology [3–9] while several model-based methods have been developed in the past decades [10–13]. Here we focus on the supervised deep learning-assisted detection of rare dynamic exocytosis events (< 1 event per frame in average) in videos. Exocytosis is a fundamental physiological process consisting in the fusion of an intracellular vesicle with the plasma membrane to release its content. It is a key step in many biological contexts from digestive enzyme secretion to the release of neurotransmitters in the synaptic cleft; and therefore represents a topic of interest for biologists over different fields. Many conventional methods of exocytosis detection have been already published [14–29] including a method partially relying on a deep learning strategy [30]. These methods detect exocytosis events visualized by an exocytic reporter protein tagged with a pH-sensitive fluorophore [31] imaged in Total Internal Reflection Fluorescence Microscopy (TIRFM). In general, a sudden peak of fluorescence intensity followed by an exponential decay of the signal (termed “puff”) is detected (Fig 1A and 1B). Unfortunately, these methods are often poorly evaluated on benchmark datasets and are not publically available. As a consequence, and despite almost 20 years of progress in exocytosis automatic detection methods, no consensual method emerged.

Here we present ExoDeepFinder, an adaptation of DeepFinder [32] (**see** **Methods**), a U-Net deep learning method, for the detection of exocytosis events in large 2D+Time volumes. DeepFinder was originally developed for the identification of macromolecules in 3D noisy cellular cryo-electron tomograms (cryo-ET) and is considered as a top-rank method, confirmed in several SHREC challenges [33]. We compared ExoDeepFinder performances to two unsupervised conventional detection methods. After demonstrating the advantage of ExoDeepFinder over the tested unsupervised methods, we evaluate its robustness to various experimental conditions. We demonstrate that our neural network approach is more robust than traditional unsupervised methods. Strikingly, the good robustness performances were achieved without re-training, demonstrating the inherent generality capacities of ExoDeepFinder. Our approach illustrates how ExoDeepFinder architecture initially designed for 3D cryo-EM data, can be transposed to another segmentation task.

## Results

### Implementation of ExoDeepFinder, a deep learning method for exocytosis detection

To train”ExoDeepFinder” we took advantage of our recently published large dataset [34] monitoring lysosomal exocytosis events via imaging of VAMP7-pHluorin by TIRFM. The coordinates of all exocytosis events were manually annotated by a single expert based on the characteristic “puff” signature (Fig 1A and 1B and S1 Movie). Because DeepFinder performances for cryo-ET segmentation substantially increased thanks to a multi-class strategy, we applied a similar approach here: we combined manual annotations of exocytosis events with automatic algorithm-generated annotations (termed ATLAS) of docked vesicles at the plasma membrane that form bright foci without fusion with the plasma membrane [35] (**see** **Methods**) (Fig 1C). Because DeepFinder is originally a segmentation method, we modeled exocytosis events as a tube with an exponentially decaying radius starting at R = 4 pixels and ending at R = 1 pixel, the length of the tube being 3 frames in the temporal dimension. This 3-frames decaying radial tube defined voxels that were assigned to the exocytosis class (Fig 1C). The centroid of the radial tube was used to convert the prediction of ExoDeepFinder segmentation predictions back into spatiotemporal coordinates (**see** **Methods**).

**Fig 2:**
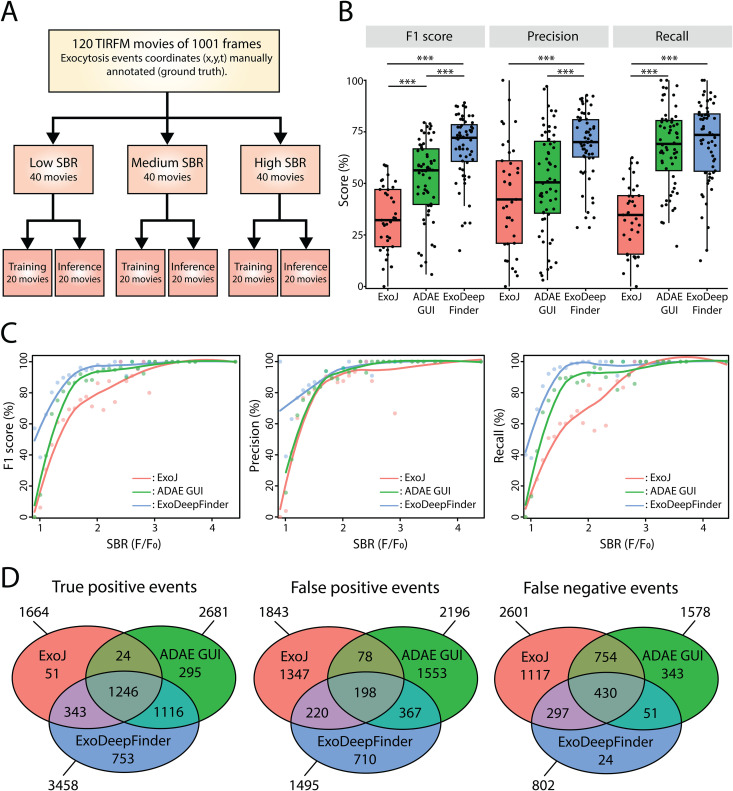
A. Organization of the dataset. The dataset is composed of 120 TIRFM movies of 1001 frames. Each movie has a manual annotation of exocytosis event coordinates constituting the ground truth. The dataset is split into 3 equal subgroups according to the average SBR (=F/F_0_) of each movie. Then, each SBR group is randomly split into two equal sub-groups, one dedicated to ExoDeepFinder training and the other one to inference, i.e., ExoDeepFinder evaluation. B. Comparison of ExoDeepFinder performances with ExoJ and ADAE GUI on the same inference dataset of 60 movies. Significance has been evaluated with a Kruskal-Wallis test (p < 0.001) and pairwise comparison with a Dunn’s post-hoc test with a multiple comparison Holm correction, ***p < 0.001 (other comparison are not significant, i.e., p > 0.05). ExoJ has less number of movies analyzed (hence less total number of events) because the analysis cannot be performed for 30-40% of the data (see method). ExoDeepFinder was trained on the total 60 movies of the training dataset (model all). C. Performances evaluated by bins of single event SBR (=F/F_0_). The solid line is a loess (with a span of 0.75). D. Venn diagram of TP, FP and FN events. Numbers indicated are the absolute counts in each category.

We hypothesized that ExoDeepFinder performances would depend on the quality of the images. Hence, we used Signal-to-Background Ratio (SBR) as a measure of image quality. SBR can be defined in two ways for our TIRFM movies, i) as a classic ratio between cell signal over background and ii) as a ratio between the local fluorescence intensity F just after the vesicle fusion with the plasma membrane and the fluorescence intensity $F_{\text{0}}$ before this peak. These two SBR did not correlate (S1A Fig). We relied on the second SBR, because it directly characterizes single exocytosis events and is the usual feature detected by former algorithms. We constituted a dataset of 120 TIRFM movies (for a total of 20 567 exocytosis events) of 1001 frames (about 6 minutes) that we divided into three groups based on their average SBR (Fig 2A). Then we split randomly each SBR group into two sub-groups, one dedicated to training of the network and the other one to its evaluation (Fig 2A). We made our dataset entirely available (**see** **Methods**).

We trained ExoDeepFinder on the three sub-datasets (low, medium, and high SBR values), on different combinations of these three datasets (Subsets A to E) (S1 Table) and on the full training dataset (i.e., 60 movies). ExoDeepFinder performances for each training set were evaluated in terms of F1-score, Recall and Precision over the whole inference dataset (*i.e.,* 60 movies). The precision measures the likelihood that a detected event is an actual exocytosis event while the recall measures the sensibility of the network, i.e., the likelihood that an actual exocytosis event will be detected. The F1-score is the harmonic mean of the precision and the recall. As commonly observed for neural network approaches, the best performances were achieved with the training over the full training dataset with 67.64% for F1, 70.07% for Recall and 68.75% for Precision (Table 1). These results demonstrated that the best performances were achieved when a broad diversity of events were presented to ExoDeepFinder during the learning phase.

**Table 1:** Performances of ExoDeepFinder for the different training datasets defined in S1 Table. Performances has been evaluated over the 60 movies dedicated for inference. Values are mean ± SD.

	F1-score (%)	Recall (%)	Precision (%)
All	67.64 ± 15.91	70.07 ± 19.81	68.75 ± 16.57
Subset A	54.77 ± 20.50	52.30 ± 23.02	64.65 ± 22.13
Subset B	61.68 ± 20.06	62.75 ± 23.65	65.94 ± 20.35
Subset C	63.62 ± 19.82	63.95 ± 23.68	69.60 ± 20.30
Subset D	62.67 ± 19.39	62.51 ± 23.58	69.67 ± 20.23
Subset E	64.51 ± 19.93	62.95 ± 22.96	70.96 ± 19.51
Low SBR	62.19 ± 18.83	69.91 ± 21.23	61.54 ± 22.20
Medium SBR	58.26 ± 20.22	70.05 ± 23.07	58.31 ± 24.74
High SBR	60.37 ± 21.18	58.26 ± 23.16	68.67 ± 22.70

Additionally, we evaluated how the multiclass strategy of ExoDeepFinder impacted performance. Interestingly, the addition of ATLAS multiclass annotations increased the precision but decreased the recall score (S1B Fig). Thus, whereas the likelihood to detect an actual exocytosis event increased (decrease of false positives), the sensibility of the detection decreased (increase in false negatives). Because false positives are often judged more problematic in biological data than false negatives, we conserved the multiclass strategy in the rest of the paper.

### ExoDeepFinder demonstrates better performances in exocytosis detection than unsupervised methods

Next, we compared the performance of ExoDeepFinder to the two unsupervised, conventional exocytosis detection methods that are publically available i) ExoJ [28] and ii) ADAE GUI [26,27].

ExoJ detection method relies on a first step of spot detection by applying the “à trous” wavelet transform similarly to the exocytosis detection method proposed by Yuan et al. [21]. Secondly, spots are tracked using the nearest-neighbour method, and finally, fusion events are predicted according to a peak of fluorescence followed by an exponential decay. ExoJ has free parameters (**see** **Methods**) that substantially impacted performance on our dataset: i) the wavelet scale σ plays a role in the spot detection step, ii) the minimal R² tolerated in fluorescence decay fitting, iii) exocytosis spot radius fited with a Gaussian and iv) an intensity ratio (dF/σ), similar to F/F_0_. We screened the ExoJ performances for the aforementioned parameters (**see** **Methods**). Note that despite numerous efforts to run the analysis on different computers overnight and with sub-parts of the movies, ExoJ failed to produce any results for a high percentage (30–40%) of the data (S1C Fig). We identified the best set of parameters of ExoJ as those that maximize the average of the 3 scores: σ = 5, decay R² = 0.8, radius R² = 0.7 and dF/σ = 3.8 (S1D Fig). Automatic Detection and Analysis of Exocytosis Events with Graphical User Interface (ADAE GUI) is another exocytosis detection method originally written in MATLAB also based on a tracking step by Kalman filter followed by a fluorescence variation analysis [26,27,29]. Recently, the authors proposed an amelioration called pHusion of the method written in Image-Tank to tackle some the limitations of the previous version like the difficulty to modify it to optimize parameters for the dataset of interest. This optimization of ExoJ and ADAE GUI performances for our dataset allows a fair comparison with ExoDeepFinder.

We compared ExoDeepFinder’s performances with unsupervised methods (ExoJ and ADAE GUI) over 60 movies dedicated to inference (Fig 2A and S2 Movie). ExoDeepFinder performed better than ExoJ and ADAE GUI in terms of F1-score and Precision. For Recall, ExoDeepFinder was statistically indistinguishable from ADAE GUI, but better than ExoJ (Fig 2B). Scores for ExoJ and ADAE GUI were lower than in their original publications, but in absence of a common benchmark these scores are difficult to compare.

As expected, performances increased with single event SBR and reached a plateau for a SBR > 2 (Fig 2C) highlighting that the SBR is a good predictor of task complexity. The dependency of the detection performance on SBR strongly argues for the use and development of exocytosis probes that increase contrast, including pH-sensitive probes. A weaker correlation was observed between performances and averaged SBR per cell (S2A-S2C Fig). ExoDeepFinder and ADAE GUI performances were robust over different frame rates (S2A and S2B Fig) contrarily to ExoJ (S2C Fig), which slightly correlated, with the frame rate. Importantly, ExoJ has been designed and evaluated on movies of 5Hz while our dataset is ≤ 3Hz, a difference which could partially explain the lower observed performances.

To characterize more precisely the differences in performances between methods, we examined the intersection of True Positive (TP), False Positive (FP) and False Negative (FN) events (Fig 2D). Surprisingly, the count of TP events specific to ExoJ was extremely low (even taking into account that the total number of movies analyzed was lower for ExoJ) demonstrating that it only detected obvious events also recognized by ADAE GUI and ExoDeepFinder. ExoDeepFinder had the highest specific count of TP events highlighting that learning approaches are able to detect subtle and less stereotyped events. In terms of FP events, the intersections were quite small (and could potentially represent ambiguous events or even annotation errors) demonstrating that each method has a different way to “hallucinate” events. Still, the count of FN events specific to ExoJ was high, suggesting that it missed many events and thus explaining its low performances. In contrast, the count of FN events specific to ExoDeepFinder was low, indicating that it mainly failed to detect events that were ambiguous or represented annotation errors.

We investigated how TP, FP and FN events differed in terms of SBR (defined as F/F_0_) and exponential decay lifetime. We found that TP events were characterized by a higher SBR than FP and FN events for all 3 methods (S2 Table). However, ExoDeepFinder showed the lowest average SBR among TP events suggesting that it additionally relied on other information. Similarly, long events were easier to detect, illustrated by the fact that TP events have longer (or similar) decay lifetimes for all 3 methods (S3 Table). Interestingly, ExoDeepFinder and ADAE GUI sometimes miss-interpreted events with a long fluorescent exponential decay (*i.e.,* high life-time) as exocytosis, contrarily to ExoJ which more often confused exocytosis with fast decay events (*i.e.,* small lifetime).

We also compared the methods in terms of localization precision of TP events (**see** **Methods**). ExoDeepFinder and ADAE GUI showed similar spatial localization precision, which was better than those of ExoJ (S2D Fig). In terms of temporal Precision, ExoJ and ADAE GUI had similar performances, which yet were lower than that of ExoDeepFinder (S2E Fig).

Finally, we evaluated how performance scores impacted experimental outcomes, especially exocytosis rate. We compared the exocytosis rate of the ground truth with those predicted by the different methods (S2F Fig). We estimated the average difference between ground truth and prediction, measured as Cohen’s *d* value (see methods), to evaluate potential systematic biases of prediction. We also assessed the level of noise in the predictions that was measured as the correlation between ground truth and predicted exocytosis rates. While ExoJ showed a good correlation with the ground truth exocytosis rate (r = 0.89), it still presented a significant bias, leading to an underestimation of exocytosis rate. ADAE GUI showed a smaller bias, resulting in an overestimation of the exocytosis rate, but exhibited a weaker correlation (r = 0.67). Finally, ExoDeepFinder achieved the highest correlation (r = 0.96) without any measurable biases.

### ExoDeepFinder exocytosis rate predictions are robust to drug perturbations

**Fig 3:**
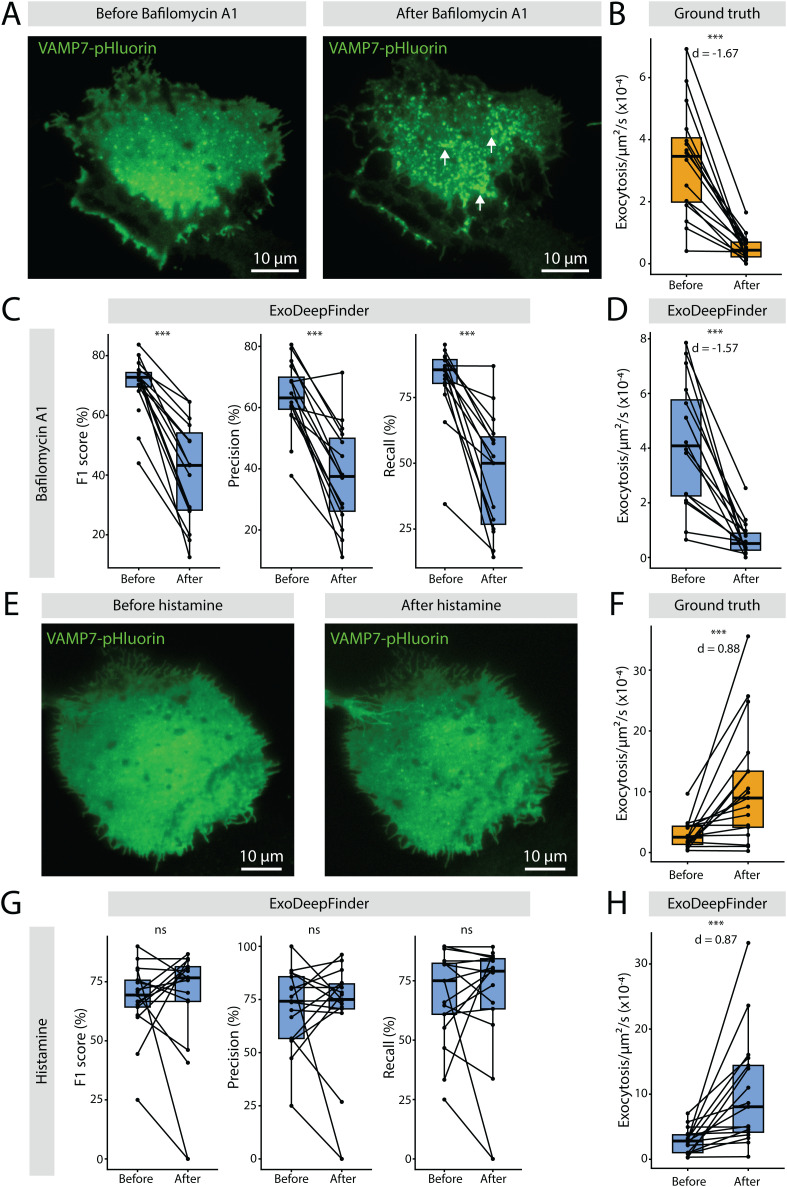
A.Representative TIRFM images of VAMP7-pHluorin in a transfected RPE-1 cell before and after Bafilomycin A1 treatment (100nM, 60min). Arrows highlight the formation of bright foci at the plasma membrane after the treatment. B. Exocytosis rate before and after Bafilomycin A1 treatment measured by manual detection (ground truth). C. ExoDeepFinder performances before and after Bafilomycin A1 treatment. Note that one point is unpaired that represents a cell with no predicted events after treatment (hence, F1-score, Precision and Recall cannot be defined). D. Exocytosis rate before and after Bafilomycin A1 treatment predicted by ExoDeepFinder. In B-D, n = 16 cells from three independent experiments with a total 3008 and 401 exocytosis events before and after drug addition, respectively, are shown. E. Representative TIRFM images of VAMP7-pHluorin in a transfected RPE-1 cell before and after histamine treatment (100 µM, cells immediately imaged after histamine addition). F. Exocytosis rate before and after histamine treatment measured by manual detection (ground truth). G. ExoDeepFinder performances before and after histamine (100µM, cells immediately imaged) treatment. H. ExoDeepFinder performances before and after histamine treatment. In F-H, n = 17 cells from three independent experiments with a total 2292 and 3201 exocytosis events before and after drug addition respectively, are shown. In B-D and F-H, significance has been evaluated with paired Wilcoxon test, ns p > 0.05 and ***p < 0.001. In B, D, F and H, effect sizes are measured with the Cohen’s *d* for paired samples. In C-D and G-H, ExoDeepFinder was trained on the total 60 movies of the training dataset (model all).

Next, we tested to what extent ExoDeepFinder performances are robust to perturbation, and to what extent it can be applied to slightly different datasets without retraining. To investigate these questions, we assessed ExoDeepFinder’s efficiency on cells treated with different drugs which interfere with secretion, without retraining the network. First, we analyzed cells before and after treatment with Bafilomycin A1, a drug impairing lysosomal pH homeostasis that leads to decreased exocytosis rate and additionally changes cell morphology by creating dynamic bright foci at the plasma membrane (Fig 3A and 3B and S3 Movie). ExoDeepFinder performances decreased significantly after the drug treatment when compared to same cells before treatment (Fig 3C). However, ExoDeepFinder was still able to estimate correctly exocytosis rate and produced a correct estimation of drug effect size (Fig 3D) even though it was exclusively trained on exocytosis events from untreated cells. Indeed, Cohen’s *d* predicted by ExoDeepFinder was -1.57, while ground truth was *d* = -1.67. We next compared ExoDeepFinder’s robustness with that of ExoJ and ADAE GUI on the same dataset. Since our objective was to evaluate generalization performances without retraining, we used the previously optimized parameters for ExoJ and ADAE GUI (Figs 1 and 2) to ensure a fair comparison. Similarly, ExoJ and ADAE GUI performances decreased (S3A and S3C Fig), but contrarily to ExoDeepFinder, ADAE GUI and ExoJ made wrong estimations of exocytosis rates, especially after treatment (S3B and S3D Fig). Both predicted an effect in the wrong direction (*d* = 0.10 for ExoJ and *d* = 0.37 for ADAE GUI) likely due to the presence of dynamic bright foci. Thus, the lower performances of detection cannot be attributed to a change in SBR (F/F_0_) (S4 Table).

Additionally, we analyzed cells treated with histamine, a drug which stimulates lysosomal exocytosis [34,36]. Contrarily to Bafilomycin A1, cell morphology and the profile of individual secretory events were almost totally preserved (including their SBR, S4 Table) following histamine treatment, and exocytosis events mainly changed in frequency (Fig 3E and 3F). In these conditions, we observed that ExoDeepFinder performances were not impaired (Fig 3G) and estimated precisely exocytosis rate as well as effect size (predicted *d* = 0.87 while ground truth *d* = 0.88) (Fig 3H). ExoJ and ADAE GUI performances were not impacted by histamine treatment either (S3E and S3G Fig). However, due to their inherent lower performances, estimated exocytosis frequency and effect size were less accurate (*d* = 0.37 for ExoJ and *d* = 0.98 for ADAE GUI) (S3F and S3H Fig). This demonstrated in real conditions that ExoDeepFinder produced better estimations of exocytosis rates and effect sizes than former methods.

### ExoDeepFinder detects exocytosis in unseen cell types and for unseen fluorescent cargos

**Fig 4:**
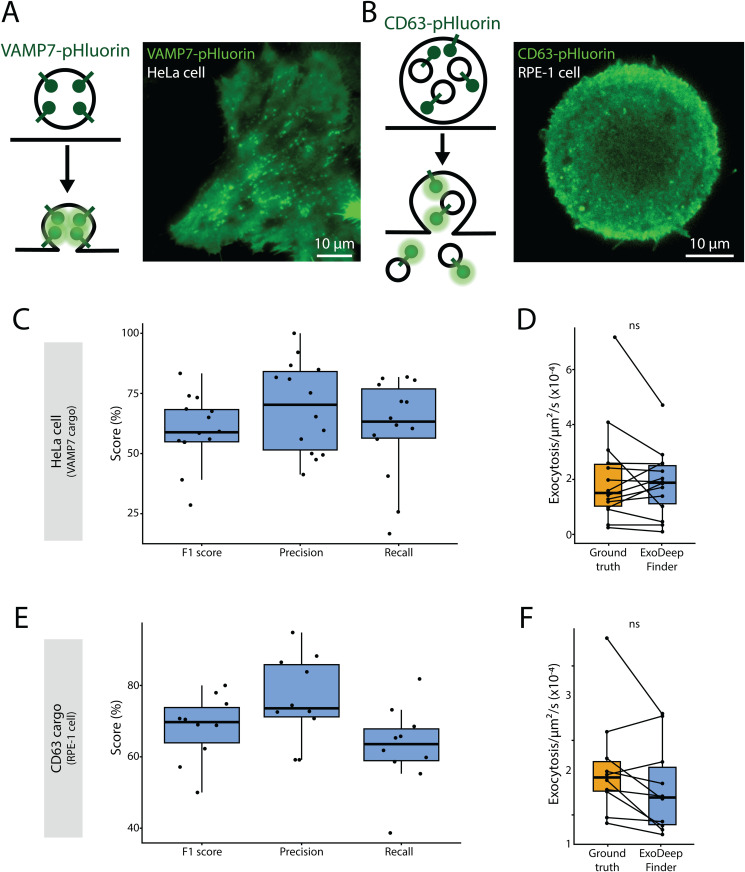
A. On the left, a schematic representation of the exocytosis of a VAMP7-pHluorin-positive vesicle. Once the vesicle starts to fuse with the plasma membrane, the pH becomes neutral and the pHluorin is no longer quenched and thus emits a fluorescence signal. On the right, a representative TIRFM image of VAMP7-pHluorin in a transfected HeLa cell. B. On the left, a schematic representation of the exocytosis of a CD63-pHluorin-positive vesicle. Once the vesicle starts to fuse with the plasma membrane, the CD63 + intraluminal vesicles are released in the extracellular medium and the pHluorin is exposed to a neutral pH and thus starts to emit a fluorescence signal. On the right, a representative TIRFM image of CD63-pHluorin-positive in a transfected RPE-1 cell seeded on ring-shaped micropattern with a diameter of 37µm. C. ExoDeepFinder performances in VAMP7-pHluorin transfected HeLa cells. D. Comparison of the exocytosis rate measured by manual detection (ground truth) and compared to ExoDeepFinder detection. In C and D, 14 cells analyzed from a single experiment with a total of 1045 exocytosis events, are shown. E. ExoDeepFinder performances in CD63-pHluorin transfected RPE-1 cells. F. Comparison of the exocytosis rate measured by manual detection (ground truth) and compared to ExoDeepFinder detection. In E and F, 10 cells analyzed from a single experiment with a total of 972 exocytosis events, are shown. In B, C, F and H, significance has been evaluated with paired Wilcoxon test, ns p > 0.05 and ***p < 0.001. In C-D and G-H, ExoDeepFinder was trained on the total 60 movies of the training dataset (model all).

We continued to evaluate the robustness of ExoDeepFinder in various experimental conditions without re-training by: i) changing the cell type but keeping the same cargo (Fig 4A) and ii) changing the cargo but keeping the same cell type (Fig 4B). Hence, we imaged VAMP7-pHluorin exocytosis events in TIRFM in HeLa cells (Fig 4A), which showed a different VAMP7 pattern than compared to RPE-1 cells on which ExoDeepFinder was trained. HeLa cells presented a different spatial organization of the bright foci and a more homogeneous membrane staining (Fig 4A). Additionally, SBR (F/F_0_) in HeLa cells was significantly lower than in RPE-1 (S4 Table). Despite this cell change, ExoDeepFinder performed similarly well in HeLa as in RPE-1 cells (Fig 4C), and the estimated exocytosis rate was statistically indistinguishable from the ground truth (i.e., manual detection) (Fig 4D). Surprisingly, ExoJ performances decreased a lot (S4A Fig): the estimated exocytosis rate decreased significantly compared to ground truth (S4B Fig). This was surprising, because part of the ExoJ validation was performed on the VAMP7-pHluorin probe expressed in HeLa cells [28]. ADAE GUI Precision decreased slightly (S4C Fig), and the predicted exocytosis rate was significantly over-estimated (S4D Fig). These differences highlight the overall importance of background staining which can differ from one cell type to another, or may be due to different exocytosis dynamics. Thus, a method that was validated in a given cell type can show loss in its performance when used in another cell type. Even though ExoDeepFinder’s performance stayed comparable in HeLa cells, the ExoDeepFinder model can be re-trained on each additional cell type, contrarily to traditional methods.

Finally, we evaluated the detection of another cargo, CD63, a transmembrane protein of the tetraspanin family that is used as a marker of the secretion of multi-vesicular bodies. Exocytosis events in RPE-1 cells were monitored with a CD63-pHluorin probe [36] (Fig 4B). The CD63 staining differed from the VAMP7 staining, and the dynamic of corresponding exocytosis events dynamic changed as well. The CD63 probe is found on endosomes as well as on intra-luminal vesicles that diffuse in 3D following exocytosis, while the VAMP7 probe remains attached to the plasma membrane and diffuses only in 2D. Hence, the CD63 probe is associated with higher SBR than the VAMP7 probe (S4 Table). Once again, we found that ExoDeepFinder performances were roughly preserved (Fig 4E), and the observed exocytosis rate was statistically indistinguishable from the ground truth (i.e., manual detection) (Fig 4F). ExoJ’s F1-score and Recall substantially increased for this dataset but the Precision value decreased (S4E Fig). Note that although CD63-pHluorin was used to validate ExoJ [28], the exocytosis rate predicted by ExoJ was underestimated, even if not significantly (S4F Fig), probably due to the low number of movies that were analyzable with ExoJ. In contrast, ADAE GUI’s F1-score and Precision decreased substantially (S4G Fig), and the predicted rate was significantly over-estimated compared to ground truth (S4H Fig). These data illustrated that changing the cargo probed can influence method performances, even when keeping the cell type constant. This conclusion emphasizes both the need for a robust detection method and a method that can be easily re-adapted to specific experimental conditions. Under this lens, deep learning approaches seem most suited to tackle this challenge.

## Discussion

In conclusion, we developed ExoDeepFinder using the U-Net DeepFinder architecture, originally designed for macromolecule detection in 3D cryo-electron cell tomograms. We demonstrated that ExoDeepFinder, trained on 60 TIRF movies and hybrid annotations, is capable of imitating expert annotations on experimental videos. Moreover, we demonstrated that it outperformed unsupervised conventional methods of exocytosis detection (Fig 2B). ExoDeepFinder achieved good performances even with smaller training datasets (S1 Table). A multi-class training of ExoDeepFinder increased its performance [32] (S1B Fig), but may require additional work for the user such as the segmentation of counter-example events. We also demonstrated that ExoDeepFinder – as other exocytosis detection methods – performs less effectively on low SBR (F/F_0_) exocytosis events, which highlights the importance for experimentalists to carefully select the frame rate, the pixel size, and fluorophores that enhance SBR, such as pH sensitive probes. A particular advantage of ExoDeepFinder is its speed to analyze large 2D+time volume data: it takes about 30 seconds (on GPU) to process a video of 300 x 300 x 1000 voxels with no parameter adjustments, contrarily to the 10–20 minutes required with the two conventional image analysis algorithms used in our benchmark, and which additionally required manual parameter calibration. The ExoDeepFinder training required 8–18 hours of computing (once for all), depending on the desired number of epochs and the GPU performance.

Furthermore, ExoDeepFinder was demonstrated to perform robustly on TIRF videos in variable experimental conditions (various cargo, cell lines, SBRs, etc.) (Figs 3 and 4) while traditional methods’ performances were perturbed by such changes (S3 and S4 Figs). For example, ExoDeepFinder was able to reliably detect exocytosis events on signals from previously unseen proteins, thus illustrating the generalization capacity of deep learning approaches. Though no method has infinite robustness, in contrast to traditional exocytosis detection methods, deep learning methods can be re-trained in addition to being fine-tuned. Hence, we endeavored to produce a user-friendly software version of ExoDeepFinder which is publicly available (**see Data and software availability**) to give users the possibility to re-train the network on their own data. This possibility to customize ExoDeepFinder to given experimental conditions is crucial, as we demonstrated how changes in cell type or cargo, for example, can affect exocytosis detection methods’ performances, especially when using traditional methods (Fig 4). Moreover, customization offers the possibility for further improvements such as the combination of several ExoDeepFinder models, and a consensus prediction based on a voting system. Another possible line of improvement would be to implement a “curriculum learning” approach via training from “easy data” to more “difficult data”, for instance using SBR. It has been demonstrated that curriculum learning improves the performances of deep learning models [37].

In conclusion, our analysis highlights the advantages of using a deep learning approach for the segmentation of dynamic processes. We show, how network architecture can be adapted from one problem (segmentation of macromolecules in cryo-EM) to another (segmentation of exocytosis events in TIRFM). We provide i) an open-source implementation of the ExoDeepFinder software, ii) the manual annotated dataset for training, and iii) the trained model for inference and fine-tuning. Detection of exocytosis events is a difficult task, because exocytosis events are rare (only few pixels in a movie) and noisy; with false positive stable bright foci highly similar to *bona fide* exocytosis event. These challenges are not specific to exocytosis detection, and we hope that the success of ExoDeepFinder’s performance will open the possibility to develop similar deep learning approaches for analysis of other dynamic processes, such as endocytosis, or the detection of blinking spots in the context of super-resolution microscopy.

## Methods

### Cell culture

hTERT-immortalized retinal pigment epithelial cell line (hTERT RPE-1) were cultivated in DMEM/F12 media (Gibco, catalog # 21041–025) complemented with 10% Fetal Bovine Serum (Eurobio, catalog # CVFSVF00–01) (without antibiotics). HeLa immortalized cell line were cultivated in DMEM high glucose (4.5g/l) media (Gibco, catalog #11965092) (without antibiotics). Cells were maintained at 37°C with 5% CO_2_ in a humidified incubator.

### Transfection

Cells were transfected with the following constructs: VAMP7-pHluorin [38] and CD63-pHluorin (addgene plasmid #130901) [36]. Moreover our dataset includes VAMP7-pHluorin transfected cells co-transfected with mCh-Rab6A or Paxillin-mCh [39]. Cells were transfected with 800ng (or 2 × 400ng for co-transfection) of DNA using the JetPrime kit (Polyplus). Cells were imaged 24h after transfection.

### Drug treatments

Cells were treated with Bafilomycin A1 (MedChemExpress, catalog # HY-100558, 100nM) for 1h before imaging or treated with histamine (Merck, catalog # H7125, 100µM) and immediately imaged. For all drug conditions, a paired design was used: the same cell was imaged before and after the treatment. Note that for histamine treatment only the first 60s after drug addition were analyzed, because the effect of histamine on secretion rate was immediate but transient [34].

### Micropatterning

Our dataset included some cells seeded on a micropatterned substrate. We followed the photolithography micropatterning protocol from Azioune et al. [40]. Briefly, coverslips (1.5H ThorLabs, Catalog # CG15XH1) were oxidized by plasma-cleaner (Harrick Plasma) during 5min. Coverslips were PEG-coated by incubating them on a drop of PLL-g-PEG [Surface Solutions, PLL(20)-g[3.5]- PEG(2)] (0.1 mg/mL diluted in water, 10mM HEPES, pH = 7.4) in a moiety chamber during 1h. After coating, patterns were printed using a deep UV lamp (Jelight Company Inc, catalog # 342–220) with radiation passing through a photomask (DeltaMask) during 5min. Finally, patterns were fibronectin-coated by incubating coverslips on a drop of fibronectin (Merck/Sigma, catalog # F1141) (50 µg/mL diluted in water) and fibrinogen-Alexa647 (Molecular Probes, Invitrogen, catalog # F35200) (or fibrinogen-Alexa488) (5 µg/mL) in a moiety chamber during 1h. Coverslips were conserved at 4°C in PBS.

Cell seeding on micropatterns was described in Lachuer et al. [41]. Briefly, coverslips were maintained in magnetic chamlides for live imaging. ~ 200,000 trypsinized (Thermo Fisher, catalog # 12605010) cells were added in the chamlide chamber. After 10min incubation in 37°C incubator, cells were attached to the substrate. Cells were washed using DMEM/F12 media with 20mM HEPES (Gibco, catalog # 15630–056) with 2% penicillin/streptomycin (Gibco, catalog # 15140–122). Cells were incubated at least 3h in the incubator until full spreading on the micropattern. Cells were imaged the same day. Two different geometries of micropatterns were used: i) ring-shaped micropatterns with a diameter of 37µm and 7µm thickness of the adhesive ring; and ii) rectangular micropatterns with 9x40µm dimensions.

### TIRFM

Non-patterned cells were seeded in fluorodishes (World Precision Instrument) coated with fibronectin (or Poly-L-Lysin (PLL) (Merck P4707) for some cells of the dataset). Media supplemented with 20mM HEPES was used for imaging. The acquisition was made using an inverted Nikon TIRFM equipped with an EMCCD camera (efficiency 95%) with a 100 × objective (pixel size = 0.160 µm) with the 491nm laser. Time-lapse of VAMP7/CD63-pHluorin was acquired with a theoretical frame rate of one image every 300ms during 5min. Frame rate was set according to the half-life of exocytosis events. Due to microscopic device delay, the actual frame rate has been computed using the computer time of saved files.

### Statistical analysis

All statistical analyses were made with R [R Core Team (2021)]. The number of cells and the number of independent repetition is indicated in the legend. Since our cells were mostly isolated when imaged, we performed only single cells analysis, each cell was considered as independent, setting the sample size. The statistical test used was indicated in the legend. Tests were always conducted in a two-sided manner and a multiple comparison correction was applied when needed. To make no hypothesis about data normality we only applied nonparametric tests (Kruskal-Wallis test with Dunn’s post-hoc test realised (with a Holm-Bonferroni correction) with the dunn.test package) and applied pairing when possible (paired Wilcoxon test). Finally, correlation was measured by Pearson correlation coefficient and tested with a t-test or with a Spearman correlation coefficient for non-linear trends.

Effect size of paired data was analyzed with Cohen’s *d*. Briefly, a value of exocytosis rate x was obtained before and after treatment for each cell. The pairwise difference ${\Delta x}_{i}$ for cell i was computed as $x_{after,i}-x_{before,i}$. The Cohen’s *d* was computed as:


$$d=\frac{\overset{―}{\Delta x}}{s_{\Delta x}}$$

With $\overset{―}{\Delta x}$ the average of the pairwise differences ${\Delta x}_{i}$, and $s_{\Delta x}$ the standard-deviation of the pairwise differences ${\Delta x}_{i}$.

### Image analysis

#### Manual exocytosis detection (Ground truth).

Exocytosis events were detected manually based on the visual recognition of a sudden burst of intensity signal followed by an exponential decay. However, this task is prone to error due to the size of the dataset and the ambiguity of some events. Our dataset probably includes FP and FN. However, these errors are minor, reflect real situations and did not impair ExoDeepFinder training.

#### Exocytosis detection with ExoJ.

ExoJ is an open-source imageJ pluggin (https://www.project-exoj.com) [28] based on the “à trous” wavelet transform (see Yuan *et al.* [21]) followed by spot tracking using the nearest-neighbour method. Fusion events are predicted according to a peak of fluorescence followed by an exponential decay. We used version 1.09 (2022-09-28) of ExoJ for our analysis. After several trials on out dataset, we determined values of the free parameters that produce satisfying results.

*Vesicle detection step:* the minimal and maximal vesicle radii were set to 2 and 5 pixels, respectively (pixel size is 160 nm). We did not apply photobleaching correction, because it created artifacts as mentioned by the authors.

*Tracking step:* the spatial searching range was set to 2 pixels. The temporal searching depth (gap closing) was set to 1 frame and the minimal event size was set to 2 frames. These values are given by default and were appropriate to process our data.

*Event detection:* we set to 5 the minimal number of points for fitting procedure, 4 the number of expanding frames (pre and post peak). We did not specify limits for the number of frames for decay, the estimated radius limit nor the maximum displacement.

The remaining parameters (wavelet scale σ, detection threshold dF/σ, minimal R² for decay and minimal R² for estimated radius) were more difficult to determine. Screening was limited by the fact that ExoJ is not a fully automatized algorithm. We run the analysis of the entire training dataset (60 movies) for 4 wavelet scales σ∈{2,3,5,10}. We applied a detection threshold dF/σ of 2, a minimal R² for decay of 0.7 (except for wavelet scale of 2 where we used 0.85 to reduce the number of movies where analyse could not be completed), and a minimal R² for radius of 0.6. Results table associates each event to dF/σ and two R², therefore higher thresholds could be tested *a posteriori* contrarily to wavelet scale that had to be set *a priori* (see S1C Fig). We emphasize on the fact that a substantial part of the movies could not be analyzed (S1B Fig); the event identification step never completed despite overnight computation. We tried different computers, different versions of ImageJ and to cut the movies without success. Most of the movies, which could not be analyzed, contained a vesicle detection step that was severely wrong, showing a high number of FPs that were even detected at high wavelet scales.

#### Exocytosis detection with ADAE GUI.

Urbina *et al.* proposed an exocytosis detection method based on the h-dome transform, tracking with Kalman filtering and fitting of fluorescence intensity by an exponential decay [26]. Authors adapted the method in a new and open-source version of the MATLAB code called ADAE GUI [27] and we used it in our experimental study. The method detects exocytosis events using Difference of Gaussians (DoF). This algorithm, often applied for spot detection, consists in subtracting the result of the image filtered with a Gaussian filter of parameter σ_1_ from the result of the image filtered with a Gaussian filter of parameter σ_2_ > σ_1_. The resulting image presents sharp maxima at the spot locations. The first step of the algorithm consists in creating the mask of the cell. We skipped this step and put a unique cell mask for the whole movie as input, generated by thresholding the intensity (ImageJ plugin) of the first frame, because cell movements are minor in our 5–7min movies. This mask typically avoided false detection on the cell borders due to small movements of the cell. Next, a background subtraction (constant value defined as the average intensity outside the cell) was applied on the whole movie. Each frame was then analyzed separately, and two pre-processings were applied: an adjustment of the intensity histogram to match the histogram of the first frame (to correct potential bleaching) and a subtraction of the median projection of the five previous frames (to highlight exocytosis events and remove stationary objects and noise). DoG at several scales (9 scales in our case) was computed, with an initial σ of 1 pixel (σ is multiplied each time by 1.25). The selected image was the median projection of the 9 images. Applying this workflow to all frames, we obtained a movie with highlighted nonstationary spots. The maximum value in each frame was computed and the median of this set of values was defined as the threshold. If a spot had at least one pixel above this median value, it would be considered as an exocytosis event. Finally, a tracking algorithm (based on Kalman filter) was applied to avoid counting several times the same exocytosis event, if present in several successive frames. The exocytosis events were given as a CSV file containing the positions (x,y,t) of particles in the movie.

#### Computation of performance scores.

The performances of the 3 methods (ExoDeepFinder, ADAE GUI and ExoJ) were evaluated in the same way through the comparison of 3D coordinates from predicted events with the ground truth. For each movie, a cell mask was generated (ImageJ intensity-based thresholding on the first frame of the movie) and events outside the masks were removed before evaluation. Moreover, for the comparison of the 3 methods (Fig 1) (but not for robustness comparison) (Fig 2), events in the 25 first and last frames were removed to avoid any bias due to border effects. User can easily apply the same exclusions in real data. A predicted event was classified as a TP if the spatial distance Δx to the nearest ground truth event is Δx≤4.5 pixels and the temporal distance Δt is −2≤Δt≤5 frames (positive sign indicates predicted event after ground truth peak). These distances Δx and Δt for TP events are reported in S2D–S2E Fig. In the rare cases when several ground truth events matched the predicted event, the nearest spatial neighbor was chosen. All predicted events, which were not classified as TP, were classified as FP and the number of FN was computed as #FN=N−#TP with N the number of events in the ground truth list. The F1-score, Precision and Recall were computed as follows:


$$F\text{1}-score=\frac{\text{2}\times \#TP}{\text{2}\times \#TP+\#FP+\#FN}$$

$$Precision=\frac{\#TP}{\#TP+\#FP}$$

$$Recall=\frac{\#TP}{\#TP+\#FN}$$

If #TP=0, #FP=0 and #FN=0 (once case in the Bafilomycin A1 dataset), these scores could not be computed.

#### Lifetime and F/F_0_ quantification.

Average fluorescence intensities were measured in a window of 7x7 pixels (1.12x1.12µm) centered on the exocytosis event during 16 frames (~5s) starting from peak intensity. The fluorescence intensity was normalized by the peak intensity. The intensity profile of all events in a cell was averaged. This averaged intensity decay was fitted with a single exponential function:


$$I(t)=Ae^{-t/t_{\text{1}/\text{2}}}+B$$

With t_1/2_ the half-life (reported in S3 Table). The fitting was performed with R using the minpack.lm function nlsLM().

The SBR ratio F/F_0_ (reported in S2 Table) was computed as the ratio between the mean intensity in a 7x7 pixels (1.12x1.12µm) window centered on exocytosis spot at the peak of fluorescence and 5 frames before. Contrarily to lifetime, a F/F_0_ SBR was computed for each event and not averaged per cell.

#### Venn diagram.

The construction of Venn diagrams (Fig 2D) started with the classifications of all events as TP, FP and FN for ExoDeepFinder, ExoJ and ADAE GUI. To be in intersection area, events associated to different detection methods were separated with Δx≤4.5 and |Δt|≤7 frames.

### ExoDeepFinder

#### Supervised deep learning for object detection in biological microscopy.

DeepFinder is an object detection method for 3D volumes that has originally been developed for detecting macromolecules in cryo-ET [32]. It is based on image segmentation using a 3D convolutional neural network, and is trained in a supervised manner. The network architecture is a U-Net [2] and is described in [32]. After segmenting the objects of interest, the coordinates of the object centroid (*i.e.,* center of mass) are retrieved by applying spatial clustering (mean-shift) to the segmentation map. Special features of ExoDeepFinder include an efficient implementation for handling large 3D datasets and strategies for dealing with rare classes. Furthermore, for training purposes, the user only needs to provide annotations in the form of a position list (*i.e.,* coordinates of the objects centroid). ExoDeepFinder includes methods for converting the position list into an approximate segmentation map (to be used as training targets). The software is implemented in Python, is based on the tensorflow package, and is available at: https://github.com/deep-finder/tirfm-deepfinder.

#### Custom optimization hyper-parameters.

To adapt DeepFinder (designed for cryo-ET) to TIRF microscopy, the distinctive characteristics of these image modalities needed to be considered. Cryo-ET data is a volume with 3 spatial dimensions, whereas TIRF data is a volume with 2 spatial dimensions and a temporal dimension (*i.e.,* an image sequence). Therefore, we limited data augmentations to the (*x,y*) plane, like random rotations and mirroring. Furthermore, from the model architecture perspective, we only applied maxpooling in the (*x,y*) dimensions. Since an exocytosis event typically lasts only 2–3 frames, it was essential to maintain a good temporal resolution. Finally, in terms of normalization, instead of using z-standardization (0-mean, 1-std) as used for cryo-ET, here we chose quantile normalization (so that the 1st percentile is aligned to value 0, and the 99th to value 1), because we noticed performance improvement for some image sequences.

#### Generating segmentation maps from position lists.

To train ExoDeepFinder, only the objects position (centroid coordinate) needed to be annotated. Segmentation maps for training were then generated by placing an (*x,y,t*) shape model at annotated positions. The luminescence of an exocytosis is isotropic in the (*x,y*) plane (*i.e.,* a disc) and has an exponential decay in *t*. Therefore our (*x,y,t*) shape model was a tube of 3 frames with an exponential decaying radius (starting at R = 4 pixels, then R = 2 and ending at R = 1 pixel).

#### Handling rare events.

The class imbalance is severe in our target case study: most image sequences have less than 0.1% of pixels that belong to the exocytosis class. To deal with this problem, ExoDeepFinder samples 3D patches centered on annotated positions. The proportion of exocytosis event pixels in the patch is then more favorable for achieving a successful training. In this respect, when choosing the patch size, there is a trade-off between class imbalance and detection performance. On the one hand, a small patch size results in a higher proportion of the rare class, because the patch is wrapped tighter around the object of interest. The downside is that less context is included in the model decision making. As a result, the model has a high false positive rate, as the missing context of the object is an important cue. On the other hand, a large patch size mitigates this problem, as with more context the model disposes of more information, hence a lower false positive rate. Yet, the proportion of the rare class within the patch is too low, which causes the training to fail (the cost function does not decrease and the model always predicts the over-represented class, *i.e.,* the negative class). We solve this trade-off by modulating the patch size during training, from small (83 pixels) to large (483 pixels). Starting with a small patch size ensures a good proportion of exocytosis event pixels (12.3% for 83 patches), allowing to obtain initial model weight values that will ensure the success of the subsequent training. We then increase the patch size by a factor 2 every 10k training iterations (see parameters in S5 Table), so that the model gradually incorporates more image context to its decision making, which effectively decreases the false positive rate (that is initially high for small patch sizes).

#### Enhance detection performance by providing counter-examples.

Furthermore, we noticed that ExoDeepFinder tends to confuse exocytosis events (*i.e.,* blinking spots) with docked vesicles (*i.e.,* spots with no exponential decay, whose luminescence remains constant through frames). We therefore decided to include counter-examples to the training procedure. We chose to generate these counter-examples by using the unsupervised spot detector ATLAS [24], which is avalaible at: https://gitlab.inria.fr/serpico/atlas. ATLAS software enables to detect spots in 2D fluorescence images. The spot size is automatically selected and the detection threshold adapts to the local image dynamics. ATLAS relies on the Laplacian of Gaussian (LoG) filter, which both reduces noise and enhances spots. A multiscale representation of the image is built to automatically select the optimal LoG variance. Local statistics of the LoG image are estimated in a Gaussian window, and the detection threshold is pointwise inferred from a probability of false alarm (PFA). The user only has to specify i) standard deviation of the Gaussian window and ii) PFA value. The Gaussian window must be about the size of the background structures; increasing the PFA increases the number of detections. In all our experiments, we set PFA to 0.001 and the standard deviation of the Gaussian window to 21 pixels. All connected components lower than 3 pixels are discarded. Then, we merge the ATLAS detections with the exocytosis event annotations into a multi-class segmentation map (label 0 is background, label 1 is exocytosis event, label 2 is docked vesicle). As the ATLAS detections were not supervised by an expert, they contain some FPs and FNs. The quality of these detections was nevertheless sufficient for our purpose (*i.e.,* to provide counter examples). In practice, it enhanced the detection performance for exocytosis events, and it also helped to mitigate the imbalanced class problem, as shown in [32].

#### Additional implementation details and computing times.

ExoDeepFinder has been computationally trained with the Tversky loss [42] and ADAM algorithm, chosen for its good convergence rate, by setting the learning rate to 0.0001, the exponential decay rate to 0.9 for the first moment estimate and to 0.999 for the second moment estimate. No regularization (e.g., L2 regularizer or ‘drop out’) was used for processing the datasets.

The inference stage of ExoDeeFinder is fast as it takes only 33.79 seconds using a Nvidia A100 GPU to process a movie of 1001 images of 261 x 149 pixels. The training from hybrid annotations required 17 hours and 30 minutes on a Nvidia A100 GPU.

### Datasets

The training/inference dataset is composed of 120 movies of single RPE-1 VAMP7-pHluorin transfected cells acquired in TIRFM. Part of this dataset is already published in Lachuer et al. [34,41]. All these cells are in control conditions (no drug treatment). However, this training dataset includes variation in the seeding conditions. While most of the cells are seeded on fibronectin-coated glass-bottom dish (62 movies), some of them are seeded on PLL (23 movies) or micropatterns (ring or rectangles shaped) (17 and 18 movies respectively). Moreover, some of the conditions are co-transfected, either with Paxillin-mCh (19 movies) or mCh-Rab6A (5 movies). We include this diversity in the dataset to increase the robustness of ExoDeepFinder. The histamine and Bafilomycin A1 datasets come also from Lachuer et al. [34]. The HeLa and CD63 datasets were not published elsewhere. While HeLa cells were seeded on fibronectin coated glass, CD63-pHluorin transfected RPE-1 cells were seeded on ring-shaped micropattern.

## Supporting information

S1 Movie Representative movie of a VAMP7-pHluorin transfected RPE-1 cell in TIRFM. White squares highlight some of the exocytosis events characterized by a sudden apparition of a bright spot and then the 2D diffusion of the signal. Time code is in minute:second format.
(MP4)

S2 Movie Representative movie of a VAMP7-pHluorin transfected RPE-1 cell in TIRFM showing the detection of the exocytosis events for the 3 described methods, compared with the ground truth. Each event appears 3 frames before and remains 3 frames after. Performances were compared from frame 20 (00:19) to frame 981 (16:20).
(MP4)

S3 Movie Representative movie of a VAMP7-pHluorin transfected RPE-1 cell in TIRFM before and after Bafilomycin A1 treatment (100nM, 60min). Time code is in minute:second format.
(MP4)

S4 Movie Representative movie of a VAMP7-pHluorin transfected RPE-1 cell in TIRFM before and after histamine treatment (100µM, immediately imaged after the treatment). Time code is in minute:second format.
(MP4)

S5 Movie Representative movie of a VAMP7-pHluorin transfected HeLa cell in TIRFM. Time code is in minute:second format.
(MP4)

S6 Movie Representative movie of a CD63-pHluorin transfected RPE-1 cell seeded ring-shaped micropattern (37µm diameter), in TIRFM. Time code is in minute:second format.
(MP4)

S1 Fig Analysis of ExoDeepFinder and ExoJ performances. **A**. Correlation between the two types of SBR (cell signal/background and F/F_0_) in the training dataset (60 movies). Significance of the correlation has been evaluated through a t-test and the R² is indicated. **B**. Comparison of ExoDeepFinder performances with or without ATLAS multiclass annotations. Comparison over the 60 movies of the inference dataset. ExoDeepFinder was trained on the total 60 movies of the training dataset (model all) with or without ATLAS annotations. Significance has been evaluated with paired Wilcoxon’s test, *p < 0.05 and ***p < 0.001. **C**. Fraction of inference dataset for which ExoJ analysis is possible as a function of the different wavelet scales used. **D**. ExoJ performances on the training dataset as a function of its different parameters.
(PDF)

S2 Fig Analysis of ExoDeepFinder, ADAE GUI, and ExoJ performances. **A**. Correlation of ExoDeepFinder performances with frame rate and SBR (cell signal/background and F/F_0_). Spearman correlation coefficients are indicated for each plot. **B**. Correlation of ADAE GUI performances with frame rate and SBR (signal/background and F/F_0_). Spearman correlation coefficients are indicated for each plot. **C**. Correlation of ExoJ performances with frame rate and SBR (cell signal/background and F/F_0_). Spearman correlation coefficients are indicated for each plot. **D**. Cumulative distribution of spatial localization error for TP events detected by ExoJ, ADAE GUI and ExoDeepFinder. Note that the different smoothness of the curves is due to the different strategies of sub-pixel localization. **E**. Cumulative distribution of temporal localization error for all true positive events detected by ExoJ, ADAE GUI and ExoDeepFinder. In D and E, number of events for each curve are n_ExoJ_ = 1664, n_ADAE GUI_ = 2681 and n_ExoDeepFinder_ = 3458. In B-C, ExoJ has less number of movies analyzed (hence less total number of events), because the analysis could not be performed for 30–40% of the data (**see method**). **F**. Comparison of the exocytosis rate measured by manual detection (ground truth) and compared (respectively) to ExoJ, ADAE GUI and ExoDeepFinder predictions. In F, 60 cells analyzed from 21 independent experiments (only 37 cells in 15 independent experiments could be analyzed with ExoJ). Systematic biais significance has been evaluated with paired Wilcoxon test and associated effect sizes are measured with the Cohen’s *d* for paired samples. Correlation has been measured with Pearson correlation coefficient r and significance evaluated with a t-test for correlation. ns p > 0.05, *p < 0.05 and ***p < 0.001. In A-F, ExoDeepFinder was trained on the total 60 movies of the training dataset (model all).
(PDF)

S3 Fig Analysis of exocytosis detection robustness to drug conditions. **A**. ExoJ performances before and after Bafilomycin A1 (100nM, 60 min) treatment. **B**. Exocytosis rate before and after Bafilomycin A1 (100nM, 60 min) treatment measured by manual detection (ground truth) and compared to ExoJ detection. Note that in A-B, unpaired points are due to movies for which ExoJ analysis was not possible. In A-B, n = 16 cells from three independent experiments for ground truth but only 2 cells for which analysis was possible before and after treatment. **C**. ADAE GUI performances before and after Bafilomycin A1 (100nM, 60 min) treatment. **D**. Exocytosis rate before and after Bafilomycin A1 (100 nM, 60 min) treatment measured by manual detection (ground truth) and compared to ADAE GUI detection. In C-D, n = 16 cells from three independent experiments. **E**. ExoJ performances before and after histamine (100µM, cells immediately imaged) treatment. **F**. Exocytosis rate before and after histamine (100µM, cells immediately imaged) treatment measured by manual detection (ground truth) and compared to ExoJ detection. Note that in E-F, unpaired points are due to movies for which ExoJ analysis was not possible. In E-F, n = 17 cells from three independent experiments for ground truth but only 6 cells for which analysis was possible before and after treatment. **G**. ADAE GUI performances before and after histamine (100µM, cells immediately imaged) treatment. **H**. Exocytosis rate before and after histamine (100µM, cells immediately imaged) treatment measured by manual detection (ground truth) and compared to ADAE GUI detection. In G-H, n = 17 cells from three independent experiments. In B, D, F and H, significance has been evaluated with paired Wilcoxon test, ns p > 0.05, *p < 0.05, **p < 0.01 and ***p < 0.001. In B, D, F and H, effect sizes are measured with the Cohen’s *d* for paired samples.
(PDF)

S4 Fig Analysis of exocytosis detection robustness to cell type and fluorescent cargo. **A**. ExoJ performances in VAMP7-pHluorin transfected HeLa cells. **B**. Comparison of the exocytosis rate measured by manual detection (ground truth) and compared to ExoJ detection. In A and B, 14 cells analyzed from a single experiment (all movies could be analyzed with ExoJ). **C**. ADAE GUI performances in VAMP7-pHluorin transfected HeLa cells. **D**. Comparison of the exocytosis rate measured by manual detection (ground truth) and compared to ADAE detection. In C and D, 14 cells analyzed from a single experiment. **E**. ExoJ performances in CD63-pHluorin transfected RPE1 cells. **F**. Comparison of the exocytosis rate measured by manual detection (ground truth) and compared to ExoJ detection. In E-F, n = 10 cells from a single experiment for ground truth but only 6 cells for which ExoJ analysis was possible. **G**. ADAE GUI performances in CD63-pHluorin transfected RPE1 cells. **H**. Comparison of the exocytosis rate measured by manual detection (ground truth) and compared to ADAE GUI detection. In G and H, 10 cells analyzed from a single experiment. In B, D, F and H, significance has been evaluated with paired Wilcoxon test, ns p > 0.05, *p < 0.05, **p < 0.01 and ***p < 0.001.
(PDF)

S1 Table Description of the different ExoDeepFinder training datasets in terms of absolute number of exocytosis events and number of movies. For each training dataset, the numbers of exocytosis events and movies coming from the 3 originally defined training datasets (low, medium and high SBR) are indicated. Fraction of the total numbers are indicated in brackets.
(PDF)

S2 Table SBR (F/F_0_) for each class of event (TP, FP and FN) for the different detection methods. Values are means (over all events) ± SD.
(PDF)

S3 Table Lifetime of exponential decay of exocytosis event for each class of event (TP, FP and FN) for the different detection methods. Values are means (over all cells) ± SD. Note that contrarily to SBR in S2 Table, a single lifetime for each category (TP, FP, FN) is computed per movie. Several events need to be merged to obtain a robust estimation of the decay contrarily to SBR that can be easily evaluated for each single event.
(PDF)

S4 Table SBR of the different datasets. Values are means (over all cells) ± SD. Significance has been evaluated with unpaired Wilcoxon test for comparison with the reference dataset and with paired Wilcoxon test for before vs. after comparisons, ns p > 0.05 and ***p < 0.001. The sample size N is the number of cells. Bafilomycin A1 dataset originally contains one additional cell but because these cells have no exocytosis event after the drug, SBR cannot be estimated, and the cell has been removed from the analysis.
(PDF)

S5 Table Training schedule. For each round, the parameters have been changed. The patch size determines the size of the local context. A random shift is applied to the patch sampling as a data augmentation technique. Hence, objects of interest are not always located at the center of the patch. The batch size is chosen according to the available GPU capacity.
(PDF)
