# Supplementary figures and images for: Deep learning detection of dynamic exocytosis events in fluorescence TIRF microscopy
Source: PLoS Comput Biol. 2025 Oct 14;21(10):e1013556. doi: 10.1371/journal.pcbi.1013556 (PMC12520386; doi:10.1371/journal.pcbi.1013556)

A

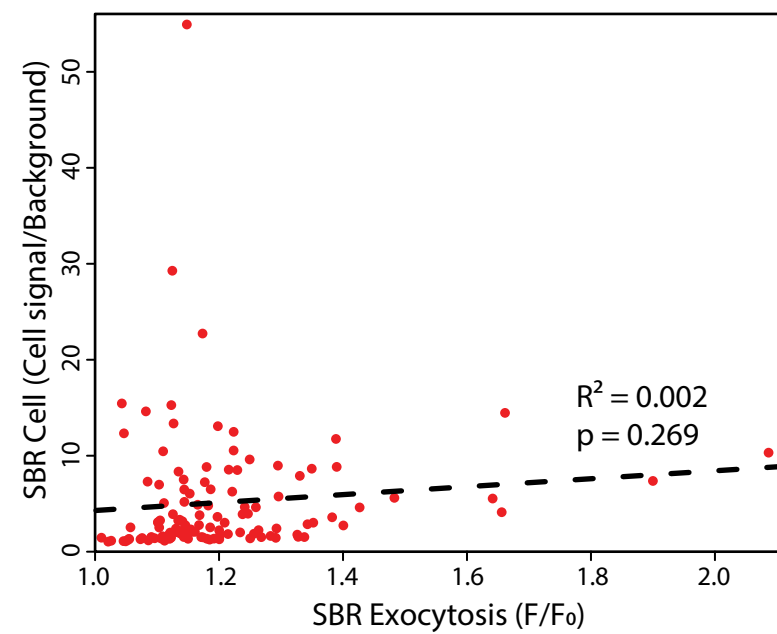

B

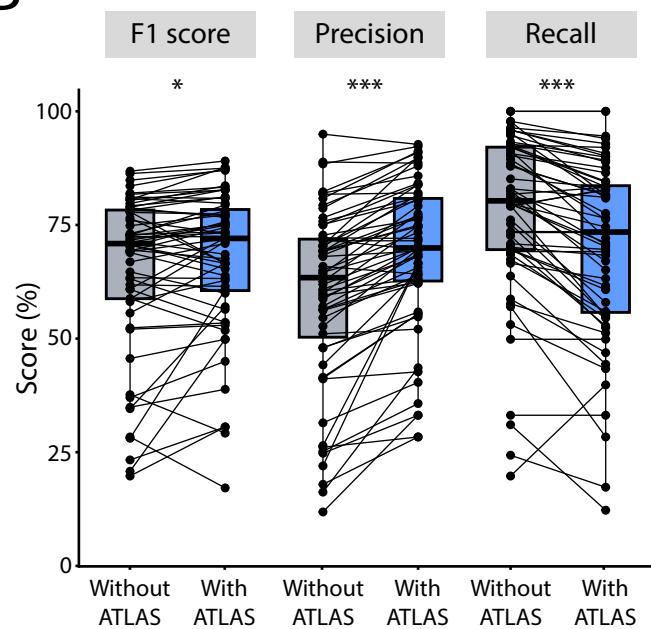

C

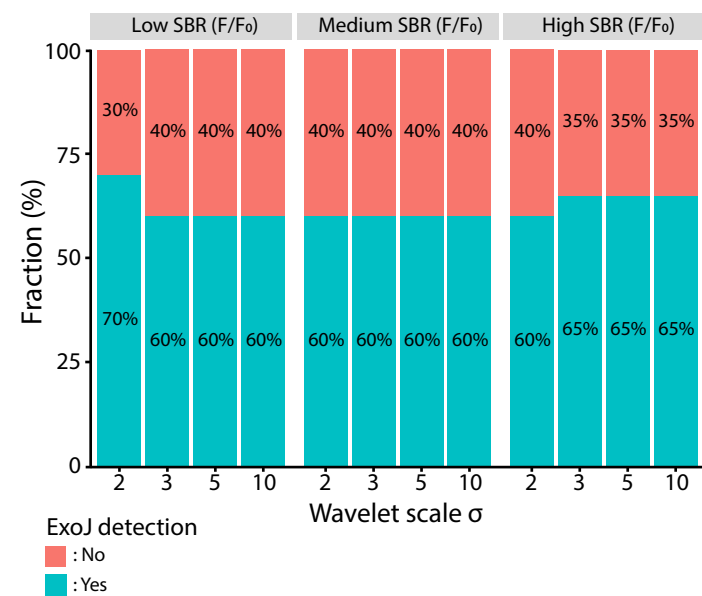

D

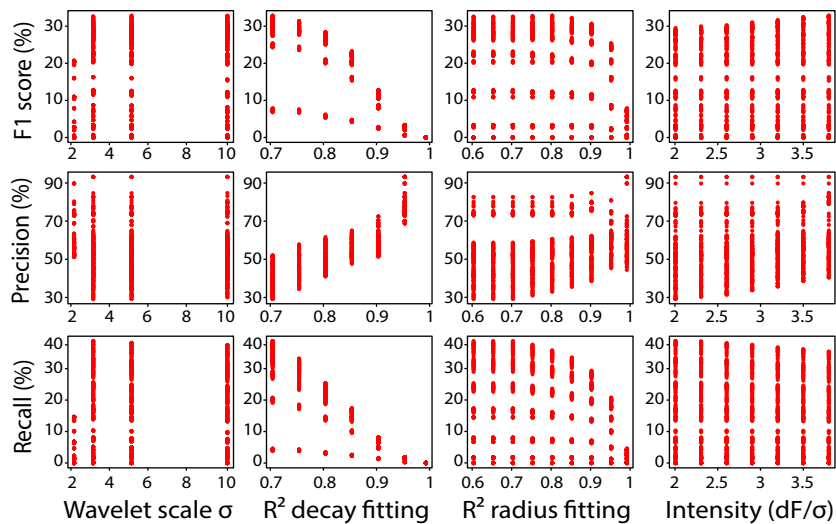

Supplement: S1 Fig — A. Correlation between the two types of SBR (cell signal/background and F/F0) in the training dataset (60 movies). Significance of the correlation has been evaluated through a t-test and the R² is indicated. B. Comparison of ExoDeepFinder performances with or without ATLAS multiclass annotations. Comparison over the 60 movies of the inference dataset. ExoDeepFinder was trained on the total 60 movies of the training dataset (model all) with or without ATLAS annotations. Significance has been evaluated with paired Wilcoxon’s test, *p < 0.05 and ***p < 0.001. C. Fraction of inference dataset for which ExoJ analysis is possible as a function of the different wavelet scales used. D. ExoJ performances on the training dataset as a function of its different parameters. (PDF) [file pcbi.1013556.s007.pdf]

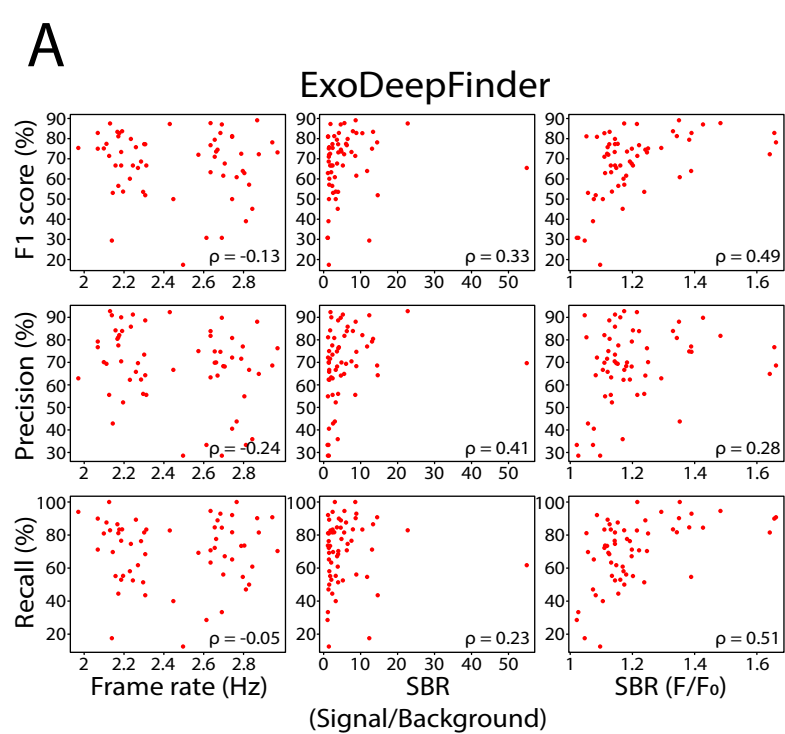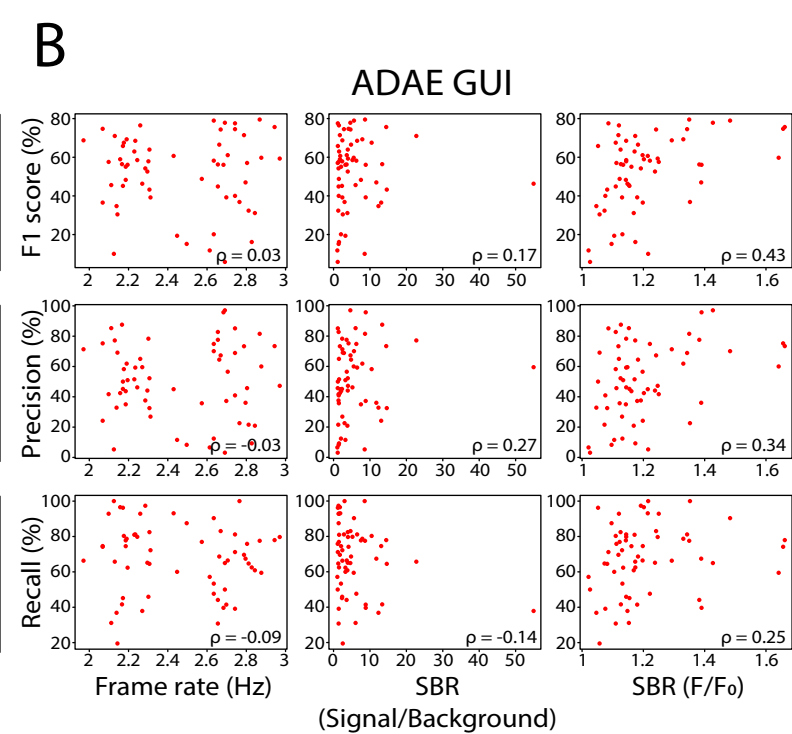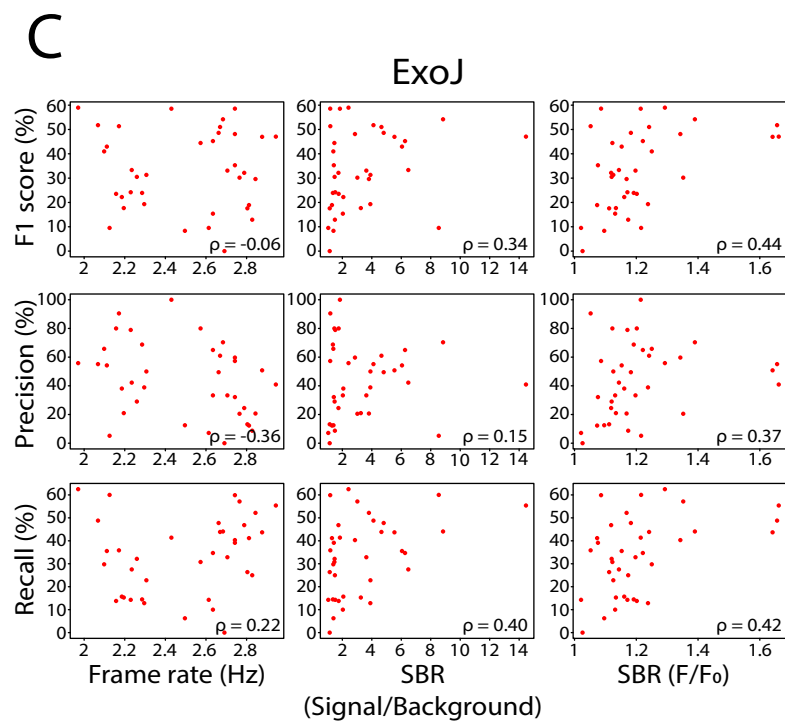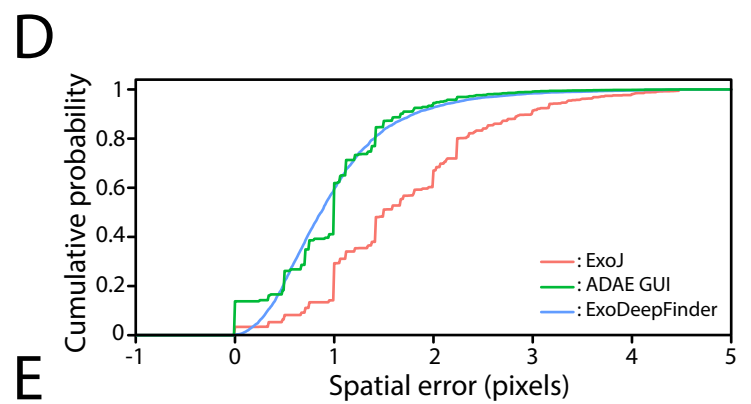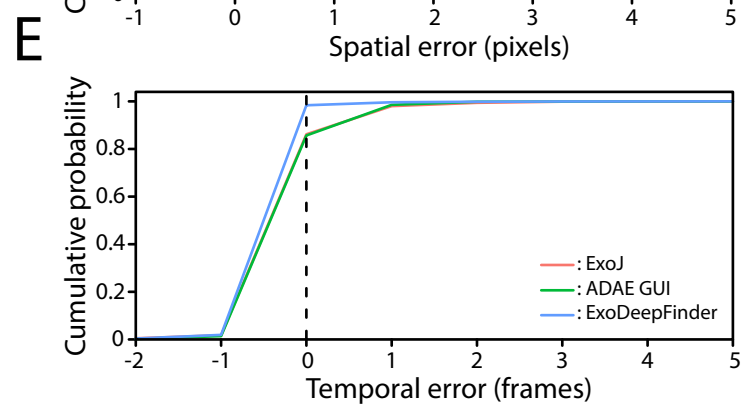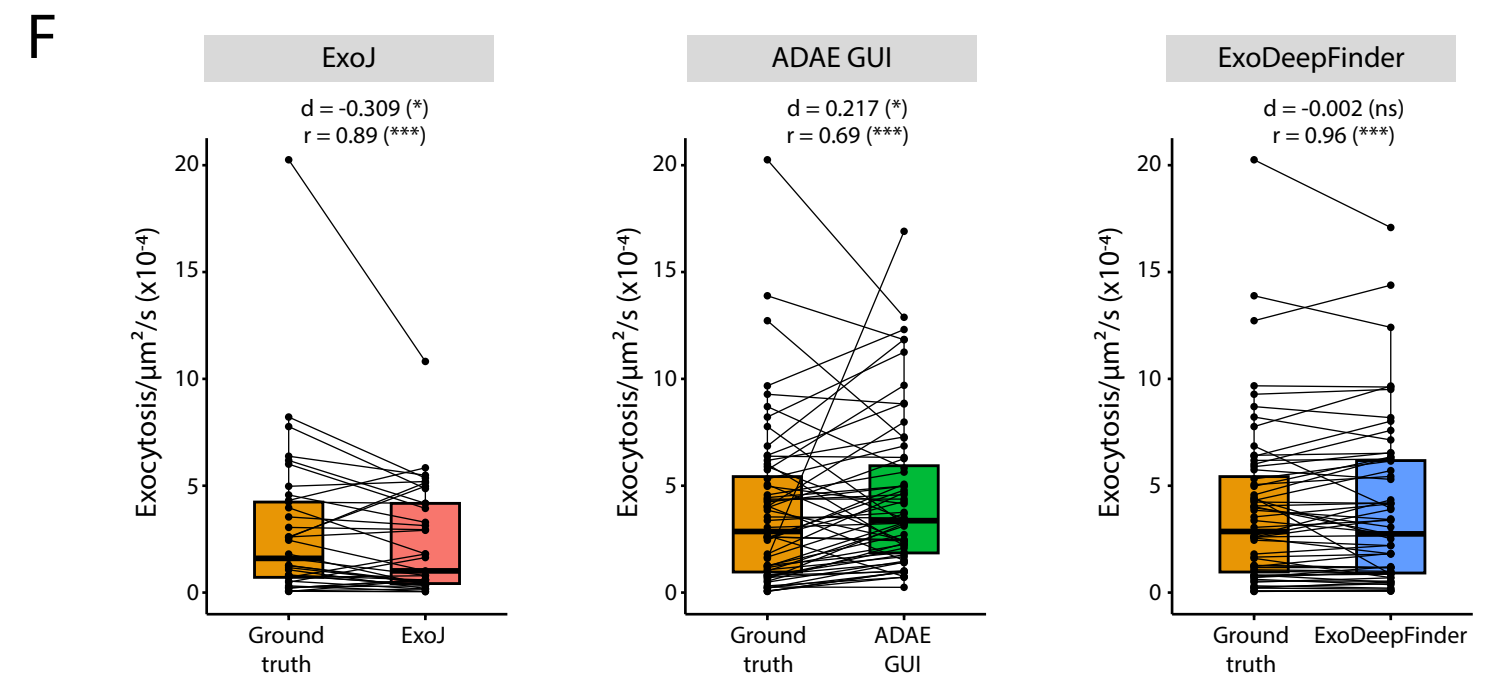

Supplement: S2 Fig — A. Correlation of ExoDeepFinder performances with frame rate and SBR (cell signal/background and F/F0). Spearman correlation coefficients are indicated for each plot. B. Correlation of ADAE GUI performances with frame rate and SBR (signal/background and F/F0). Spearman correlation coefficients are indicated for each plot. C. Correlation of ExoJ performances with frame rate and SBR (cell signal/background and F/F0). Spearman correlation coefficients are indicated for each plot. D. Cumulative distribution of spatial localization error for TP events detected by ExoJ, ADAE GUI and ExoDeepFinder. Note that the different smoothness of the curves is due to the different strategies of sub-pixel localization. E. Cumulative distribution of temporal localization error for all true positive events detected by ExoJ, ADAE GUI and ExoDeepFinder. In D and E, number of events for each curve are nExoJ = 1664, nADAE GUI = 2681 and nExoDeepFinder = 3458. In B-C, ExoJ has less number of movies analyzed (hence less total number of events), because the analysis could not be performed for 30–40% of the data (see method). F. Comparison of the exocytosis rate measured by manual detection (ground truth) and compared (respectively) to ExoJ, ADAE GUI and ExoDeepFinder predictions. In F, 60 cells analyzed from 21 independent experiments (only 37 cells in 15 independent experiments could be analyzed with ExoJ). Systematic biais significance has been evaluated with paired Wilcoxon test and associated effect sizes are measured with the Cohen’s d for paired samples. Correlation has been measured with Pearson correlation coefficient r and significance evaluated with a t-test for correlation. ns p > 0.05, *p < 0.05 and ***p < 0.001. In A-F, ExoDeepFinder was trained on the total 60 movies of the training dataset (model all). (PDF) [file pcbi.1013556.s008.pdf]

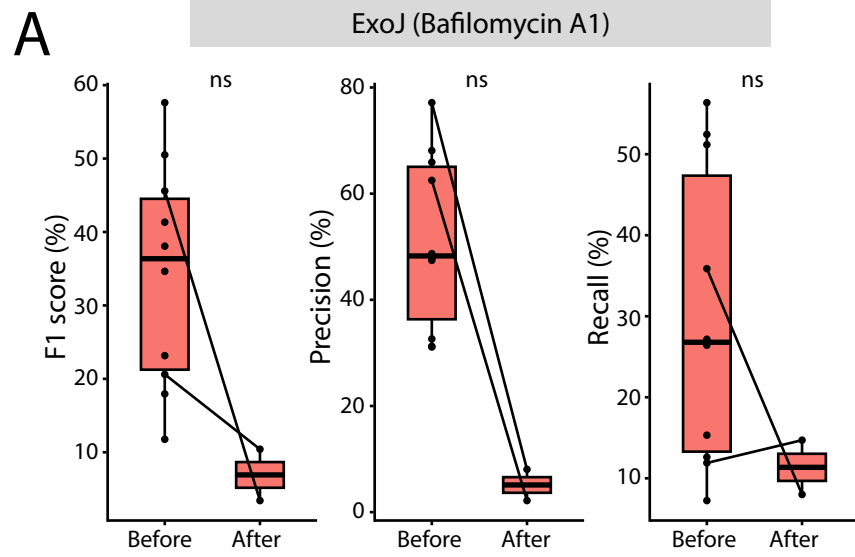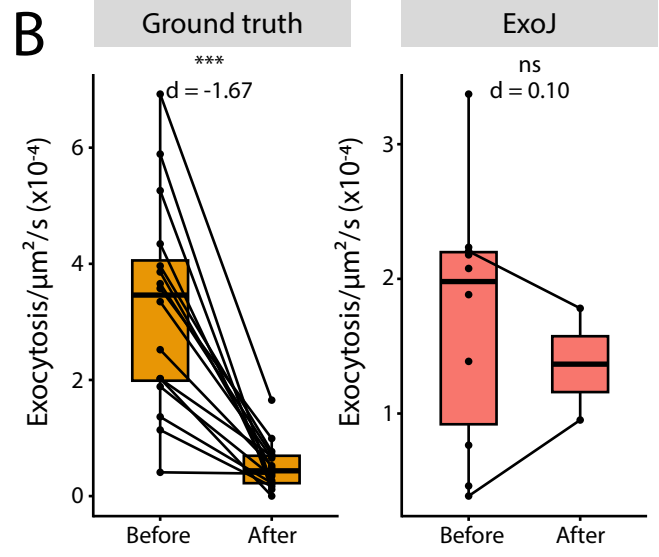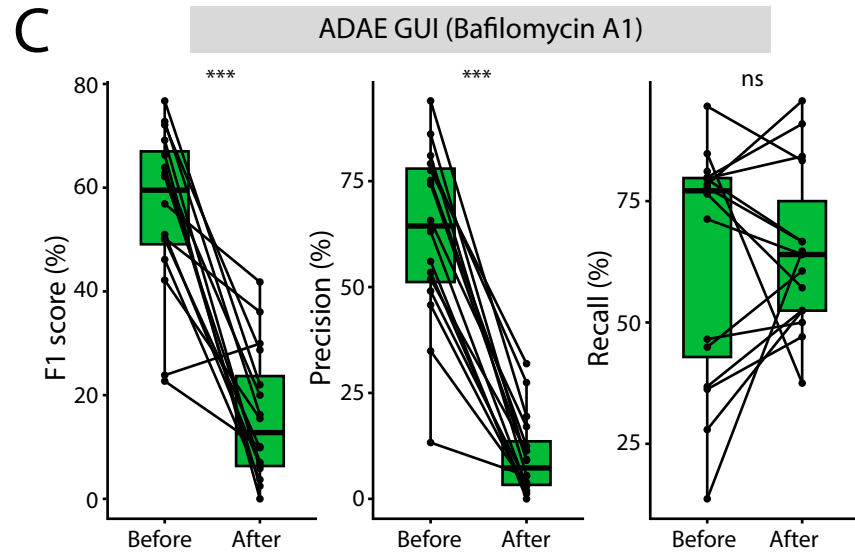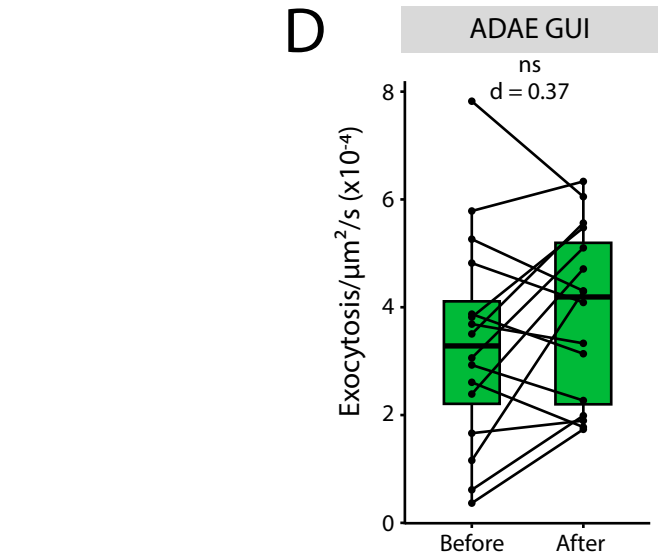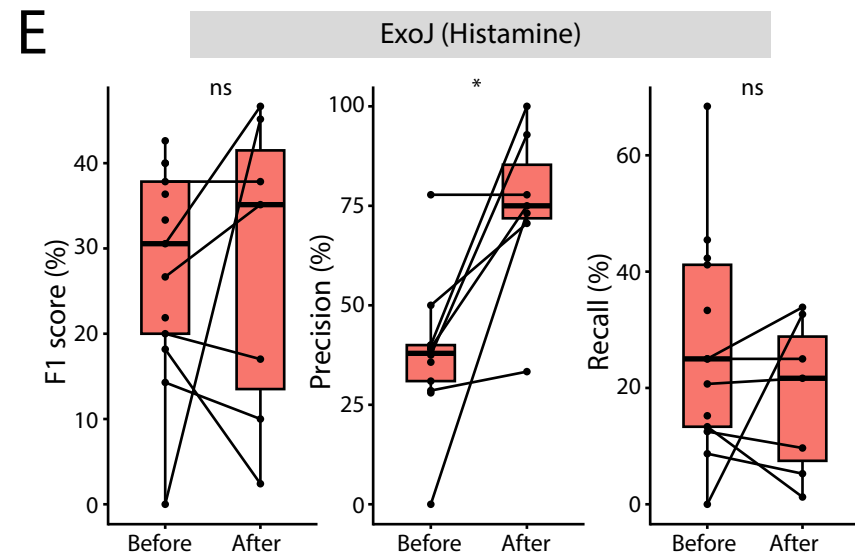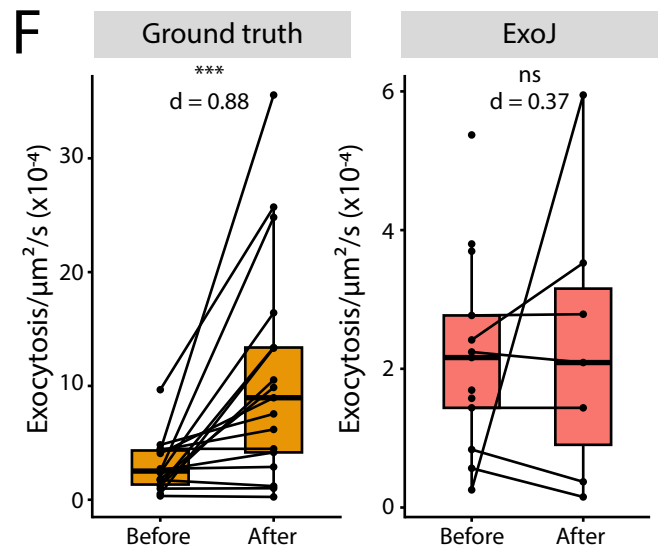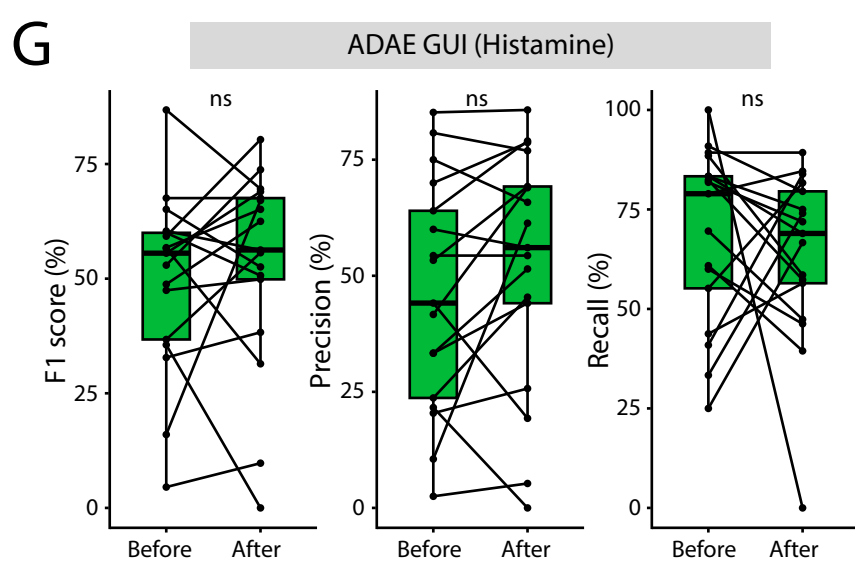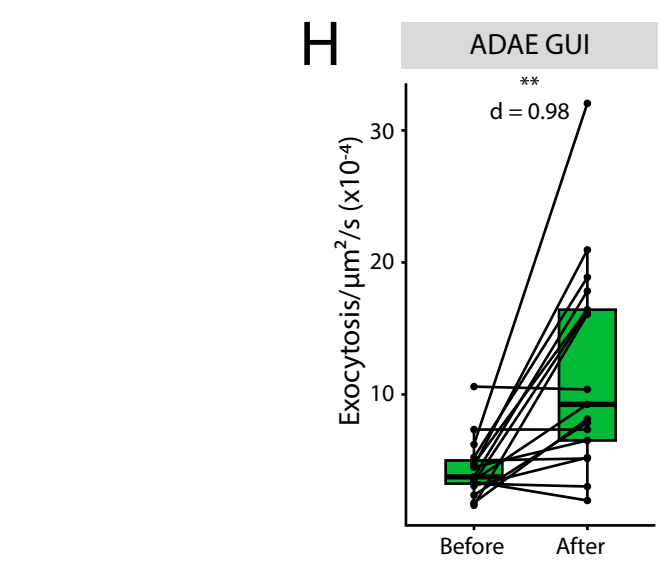

Supplement: S3 Fig — A. ExoJ performances before and after Bafilomycin A1 (100nM, 60 min) treatment. B. Exocytosis rate before and after Bafilomycin A1 (100nM, 60 min) treatment measured by manual detection (ground truth) and compared to ExoJ detection. Note that in A-B, unpaired points are due to movies for which ExoJ analysis was not possible. In A-B, n = 16 cells from three independent experiments for ground truth but only 2 cells for which analysis was possible before and after treatment. C. ADAE GUI performances before and after Bafilomycin A1 (100nM, 60 min) treatment. D. Exocytosis rate before and after Bafilomycin A1 (100 nM, 60 min) treatment measured by manual detection (ground truth) and compared to ADAE GUI detection. In C-D, n = 16 cells from three independent experiments. E. ExoJ performances before and after histamine (100µM, cells immediately imaged) treatment. F. Exocytosis rate before and after histamine (100µM, cells immediately imaged) treatment measured by manual detection (ground truth) and compared to ExoJ detection. Note that in E-F, unpaired points are due to movies for which ExoJ analysis was not possible. In E-F, n = 17 cells from three independent experiments for ground truth but only 6 cells for which analysis was possible before and after treatment. G. ADAE GUI performances before and after histamine (100µM, cells immediately imaged) treatment. H. Exocytosis rate before and after histamine (100µM, cells immediately imaged) treatment measured by manual detection (ground truth) and compared to ADAE GUI detection. In G-H, n = 17 cells from three independent experiments. In B, D, F and H, significance has been evaluated with paired Wilcoxon test, ns p > 0.05, *p < 0.05, **p < 0.01 and ***p < 0.001. In B, D, F and H, effect sizes are measured with the Cohen’s d for paired samples. (PDF) [file pcbi.1013556.s009.pdf]

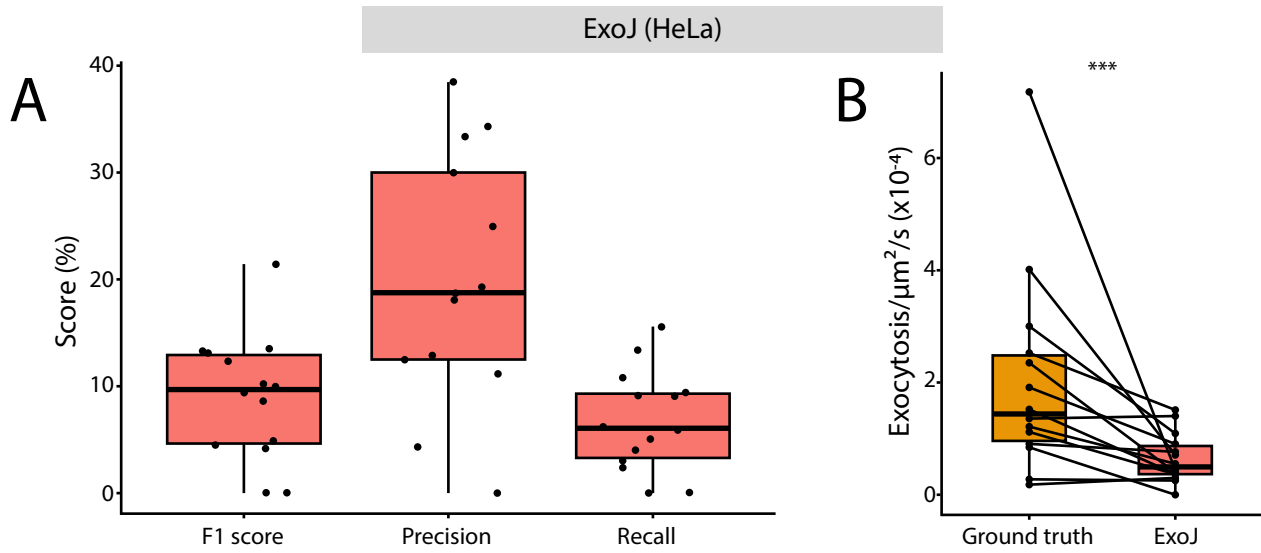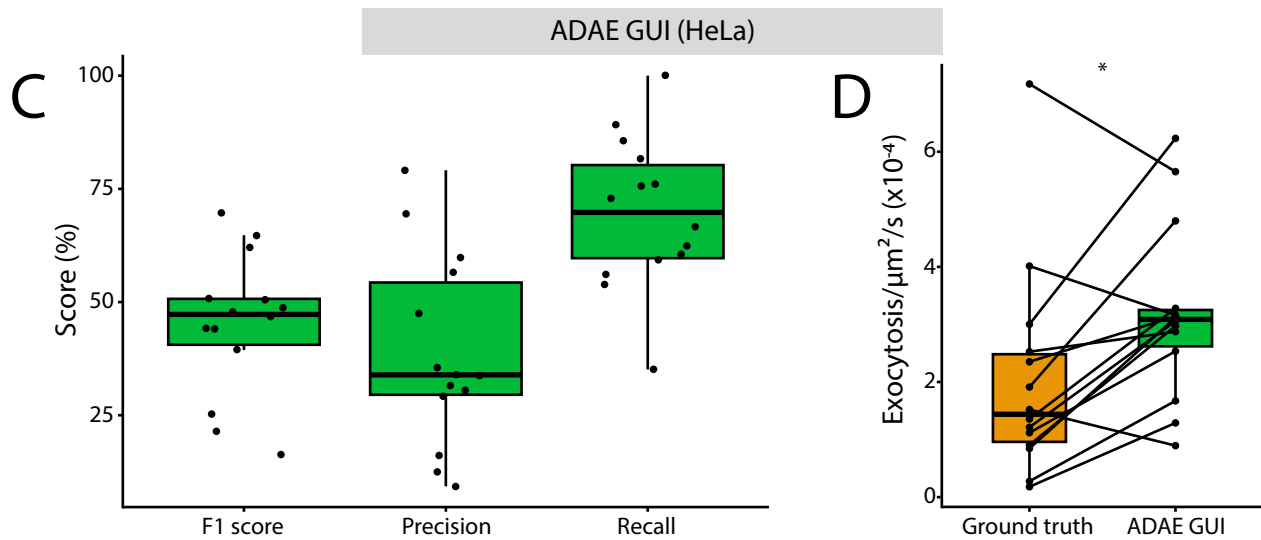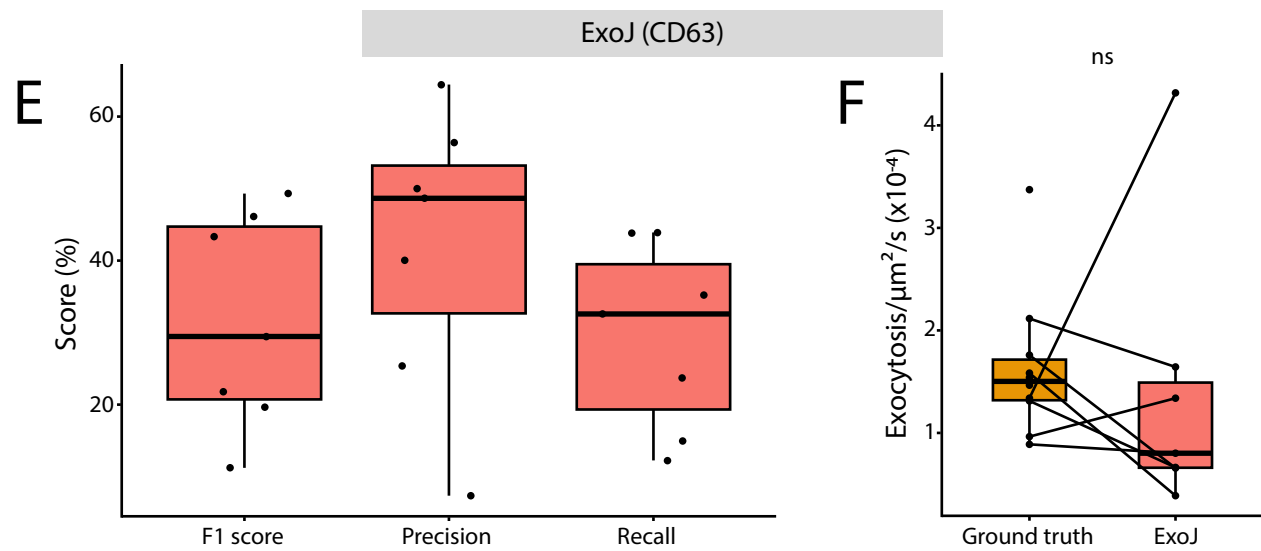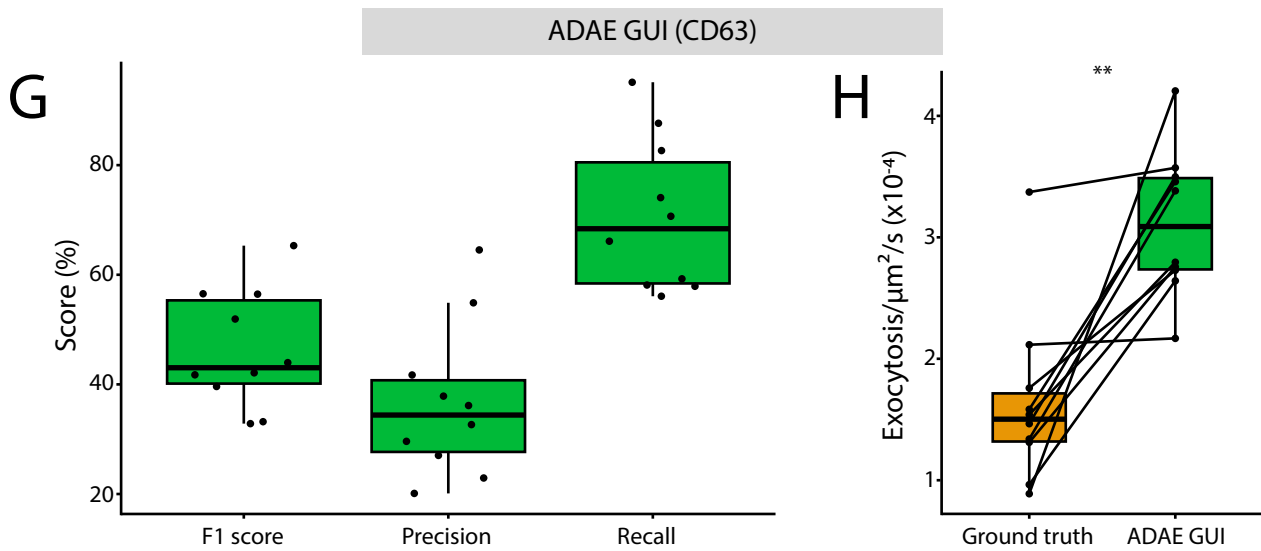

Supplement: S4 Fig — A. ExoJ performances in VAMP7-pHluorin transfected HeLa cells. B. Comparison of the exocytosis rate measured by manual detection (ground truth) and compared to ExoJ detection. In A and B, 14 cells analyzed from a single experiment (all movies could be analyzed with ExoJ). C. ADAE GUI performances in VAMP7-pHluorin transfected HeLa cells. D. Comparison of the exocytosis rate measured by manual detection (ground truth) and compared to ADAE detection. In C and D, 14 cells analyzed from a single experiment. E. ExoJ performances in CD63-pHluorin transfected RPE1 cells. F. Comparison of the exocytosis rate measured by manual detection (ground truth) and compared to ExoJ detection. In E-F, n = 10 cells from a single experiment for ground truth but only 6 cells for which ExoJ analysis was possible. G. ADAE GUI performances in CD63-pHluorin transfected RPE1 cells. H. Comparison of the exocytosis rate measured by manual detection (ground truth) and compared to ADAE GUI detection. In G and H, 10 cells analyzed from a single experiment. In B, D, F and H, significance has been evaluated with paired Wilcoxon test, ns p > 0.05, *p < 0.05, **p < 0.01 and ***p < 0.001. (PDF) [file pcbi.1013556.s010.pdf]
