# Supplementary material for: Deep learning detection of dynamic exocytosis events in fluorescence TIRF microscopy
Source: PLoS Comput Biol. 2025 Oct 14;21(10):e1013556. doi: 10.1371/journal.pcbi.1013556 (PMC12520386; doi:10.1371/journal.pcbi.1013556)
Supplement: S1 Table — For each training dataset, the numbers of exocytosis events and movies coming from the 3 originally defined training datasets (low, medium and high SBR) are indicated. Fraction of the total numbers are indicated in brackets. (PDF) [file pcbi.1013556.s011.pdf]

		Subset A	Subset B	Subset C	Subset D	Subset E	All
**# of exocytosis events**	**Total**	630	1320	2870	5732	8698	11898
	Low SBR	227 (36.03%)	515 (39.02%)	1257 (43.80%)	2522 (44.00%)	3872 (44.52%)	5458 (45.87%)
	Medium SBR	158 (25.08%)	385 (29.17%)	773 (26.93%)	1530 (26.69%)	2290 (26.33%)	3064 (25.75%)
	High SBR	218 (34.60%)	420 (31.82%)	840 (29.27%)	1680 (29.31%)	2536 (29.16%)	3376 (28.37%)
**# of movies**	**Total**	8	10	20	38	53	60
	Low SBR	4 (50.00%)	2 (20.00%)	7 (35.00%)	13 (34.21%)	18 (33.96%)	20 (33.33%)
	Medium SBR	2 (25.00%)	4 (40.00%)	7 (35.00%)	13 (34.21%)	17 (32.08%)	20 (33.33%)
	High SBR	2 (25.00%)	4 (40.00%)	6 (30.00%)	12 (31.58%)	18 (33.96%)	20 (33.33%)

Table S1. Description of the different ExoDeepFinder training datasets in terms of absolute number of exocytosis events and number of movies. For each training dataset, the numbers of exocytosis events and movies coming from the 3 originally defined training datasets (low, medium and high SBR) are indicated. Fraction of the total numbers are indicated in brackets.
