# Supplementary material for: Deep learning detection of dynamic exocytosis events in fluorescence TIRF microscopy
Source: PLoS Comput Biol. 2025 Oct 14;21(10):e1013556. doi: 10.1371/journal.pcbi.1013556 (PMC12520386; doi:10.1371/journal.pcbi.1013556)
Supplement: S2 Table — Values are means (over all events) ± SD. (PDF) [file pcbi.1013556.s012.pdf]

	**ExoJ**	**ADAE GUI**	**ExoDeepFinder**
**TP**	1.38 ± 0.35	1.33 ± 0.28	1.29 ± 0.26
**FP**	1.11 ± 0.15	1.16 ± 0.13	1.17 ± 0.14
**FN**	1.22 ± 0.19	1.16 ± 0.15	1.13 ± 0.11

Table S2. SBR (F/F_0) for each class of event (TP, FP and FN) for the different detection methods. Values are means (over all events) ± SD.
