# Supplementary material for: Deep learning detection of dynamic exocytosis events in fluorescence TIRF microscopy
Source: PLoS Comput Biol. 2025 Oct 14;21(10):e1013556. doi: 10.1371/journal.pcbi.1013556 (PMC12520386; doi:10.1371/journal.pcbi.1013556)
Supplement: S3 Table — Values are means (over all cells) ± SD. Note that contrarily to SBR in S2 Table, a single lifetime for each category (TP, FP, FN) is computed per movie. Several events need to be merged to obtain a robust estimation of the decay contrarily to SBR that can be easily evaluated for each single event. (PDF) [file pcbi.1013556.s013.pdf]

	ExoJ (s)	ADAE GUI (s)	ExoDeepFinder (s)
**TP**	2.00 ± 1.08	2.03 ± 1.12	2.05 ± 1.12
**FP**	1.17 ± 1.67	2.13 ± 2.46	1.98 ± 1.42
**FN**	1.71 ± 1.29	1.54 ± 1.77	1.35 ± 0.93

Table S3. Lifetime of exponential decay of exocytosis event for each class of event (TP, FP and FN) for the different detection methods. Values are means (over all cells) ± SD. Note that contrarily to SBR in **Table S2**, a single lifetime for each category (TP, FP, FN) is computed per movie. Several events need to be merged to obtain a robust estimation of the decay contrarily to SBR that can be easily evaluated for each single event.
