# Supplementary material for: Deep learning detection of dynamic exocytosis events in fluorescence TIRF microscopy
Source: PLoS Comput Biol. 2025 Oct 14;21(10):e1013556. doi: 10.1371/journal.pcbi.1013556 (PMC12520386; doi:10.1371/journal.pcbi.1013556)
Supplement: S4 Table — Values are means (over all cells) ± SD. Significance has been evaluated with unpaired Wilcoxon test for comparison with the reference dataset and with paired Wilcoxon test for before vs. after comparisons, ns p > 0.05 and ***p < 0.001. The sample size N is the number of cells. Bafilomycin A1 dataset originally contains one additional cell but because these cells have no exocytosis event after the drug, SBR cannot be estimated, and the cell has been removed from the analysis. (PDF) [file pcbi.1013556.s014.pdf]

		N	SBR (F/F_0)	Comparison
**Inference and training datasets (F2A)**		120	1.20 ± 0.16	
**Bafilomycin A1**	**Before**	15	1.20 ± 0.17	ns (vs. dataset of F2A)
	**After**		1.24 ± 0.16	ns (vs Before)<br>ns (vs. dataset of F2A)
**Histamine**	**Before**	17	1.20 ± 0.12	ns (vs. dataset of F2A)
	**After**		1.21 ± 0.12	ns (vs Before)<br>ns (vs. dataset of F2A)
**HeLa**		14	1.07 ± 0.02	*** (vs. dataset of F2A)
**CD63-pHluorin**		10	1.41 ± 0.06	*** (vs. dataset of F2A)

Table S4. SBR of the different datasets. Values are means (over all cells) ± SD. Significance has been evaluated with unpaired Wilcoxon test for comparison with the reference dataset and with paired Wilcoxon test for before vs. after comparisons, ns $p>0.05$ and ***$p<0.001$. The sample size N is the number of cells. Bafilomycin A1 dataset originally contains one additional cell but because these cells have no exocytosis event after the drug, SBR cannot be estimated, and the cell has been removed from the analysis.
