# Supplementary material for: Deep learning detection of dynamic exocytosis events in fluorescence TIRF microscopy
Source: PLoS Comput Biol. 2025 Oct 14;21(10):e1013556. doi: 10.1371/journal.pcbi.1013556 (PMC12520386; doi:10.1371/journal.pcbi.1013556)
Supplement: S5 Table — For each round, the parameters have been changed. The patch size determines the size of the local context. A random shift is applied to the patch sampling as a data augmentation technique. Hence, objects of interest are not always located at the center of the patch. The batch size is chosen according to the available GPU capacity. (PDF) [file pcbi.1013556.s015.pdf]

| | Training iterations | Patch size (in pixels) | Maximal random shift (in pixels) | Batch size |
|---|---|---|---|---|
| **Round 1** | 0-10k | 8 | 4 | 256 |
| **Round 2** | 10k-20k | 16 | 8 | 128 |
| **Round 3** | 20k-30k | 32 | 16 | 32 |
| **Round 4** | 30k – Until convergence | 48 | 32 | 10 |

**Table S5**. Training schedule. For each round, the parameters have been changed. The patch size determines the size of the local context. A random shift is applied to the patch sampling as a data augmentation technique. Hence, objects of interest are not always located at the center of the patch. The batch size is chosen according to the available GPU capacity.
